# A multi-epitope vaccine incorporating adhesin-derived antigens protects against *Mycobacterium tuberculosis* infection and dissemination

**DOI:** 10.3389/fimmu.2025.1707471

**Published:** 2025-11-19

**Authors:** Haoyan Yang, Xinkui Lei, Siyu Chai, Sigen Zhang, Guimin Su, Lin Du

**Affiliations:** 1 Research and Development Centre, Beijing Zhifei Lvzhu Biopharmaceutical Co., Ltd., Beijing, China; 2 Beijing Bacterial Vaccine Engineering Research Centre, Beijing, China

**Keywords:** *Mycobacterium tuberculosis*, adhesins, epitope-based vaccine, TLR2-agonist vaccine, Infection prevention, extrapulmonary dissemination

## Abstract

**Introduction:**

Adhesion to host cells is the first and essential step in *Mycobacterium tuberculosis* (*M. tuberculosis*) infection. Among adhesion molecules, the PGRS domain of PE_PGRS33 plays a critical role in invasion but is dominated by B cell epitopes and lacks sufficient T cell epitopes, restricting its capacity to induce a balanced immune response.

**Methods:**

To overcome this limitation, we employed an integrative reverse vaccinology pipeline combining computational prediction and experimental validation. Helper and cytotoxic T lymphocyte epitopes were incorporated from multiple *M. tuberculosis* adhesins as well as other virulence-associated proteins, and adjuvant sequences were systematically evaluated in silico.

**Results:**

Among three multi-epitope constructs, the Toll-like receptor 2 (TLR2)-agonist and pan HLA DR-binding epitope (PADRE)-adjuvanted vaccine (TLR2-vaccine) emerged as the most promising candidate. In murine models, TLR2-vaccine induced strong antigen-specific antibody and IFN-γ responses, significantly reduced bacterial loads following H37Ra challenge, and effectively prevented extrapulmonary dissemination.

**Discussion:**

These findings highlight the potential of adhesin-inclusive multi-epitope vaccines to elicit both humoral and cellular immunity and demonstrate how computational vaccinology can accelerate the development of targeted interventions against tuberculosis.

## 1 Introduction

TB remains a substantial global health burden, with *M. tuberculosis* as its causative agent, ranking closely behind the COVID-19 pandemic in 2022 (1). Despite extensive efforts in public health, TB continues to affect over 10 million individuals annually (1), highlighting its persistent presence and global impact. It is imperative to address the challenges faced in effective tuberculosis treatment while emphasizing the pivotal role of prevention in alleviating the disease’s global burden (2). The *Bacillus Calmette–Guérin* (BCG) vaccine is currently the only authorized prophylactic measure for TB. However, its protective efficacy is limited in the adult population. Therefore, further research and development of additional preventive measures are necessary to improve TB control strategies.

Subunit vaccines have emerged as a promising strategy due to their potent T cell and B cell stimulatory capacity (3–8). Yet, these vaccines may not provide broad protection against infection because they typically target a limited number of antigens and might cause allergic reactions without contributing to the desired immune response (9, 10). To overcome these challenges, reverse vaccinology has emerged as an effective approach. Reverse vaccinology, introduced in 2000, is an in silico approach that starts with the pathogen’s genomic sequence and ends with a list of predicted peptide candidates that require experimental verification before vaccine development (11). The first epitope-based vaccine against infectious disorders was developed in 1985 by Jacob et al. (12), and numerous others against bacteria, viruses, parasites and even cancer are currently being developed, including those for *Staphylococcus aureus*, dengue virus, human papilloma virus (HPV), visceral leishmaniasis, onchocerciasis, and breast cancer (13–18). A well-crafted multi-epitope vaccine holds promise as a tool for combating a range of diseases (19, 20). Continued research and clinical trials are necessary to evaluate the safety, efficacy, and potential of reverse vaccinology and peptide-based vaccines in TB and other disease prevention and/or therapy.

Currently, there are 16 tuberculosis vaccine candidates undergoing clinical trials worldwide (21). Most of these trials focus on therapeutic vaccines, TB prevention, and TB recurrence prevention, with few targeting *M. tuberculosis* infection specifically. However, the first line of defense against TB is actually the prevention of *M. tuberculosis* infection itself. Adhesion molecules, which facilitate the initial interaction of mycobacteria with host cells, are crucial in this context. These molecules, present on the surface of most bacteria, aid in attachment and interaction with the host during infection (22, 23).

We selected multiple adhesion molecules to develop a multi-epitope vaccine aimed at preventing *M. tuberculosis* from entering target cells. One such molecule, antigen 85B (Ag85B), is a major secretory protein of *M. tuberculosis* that binds to fibronectin on host cells (24). Ag85B is vital for *M. tuberculosis* pathogenicity, as it inhibits the formation of phagolysosomes necessary for infection clearance, allowing the bacteria to evade the host immune response (25). Clinical trial data show that while Ag85B weakly enhances humoral immunity, it significantly boosts the CD4^+^ T cell response (26, 27). Hsp65 is another key adhesion molecule that binds to CD43 (28, 29). It plays a crucial role in host cell survival mechanisms and strongly modulates immune responses based on cellular immunity (29–31). *M. tuberculosis* pili (MTP) are small adhesion molecules that interact with laminin in the host cell’s extracellular matrix, contributing to tissue colonization and potentially acting as virulence factors in *M. tuberculosis* pathophysiology (32).

In addition to adhesion molecules, we included several important *M. tuberculosis* virulence factors to broaden immune coverage. The early secreted antigenic target-6 (ESAT-6) enhances mycobacterial pathogenicity, promotes host cell cytolysis, reduces the ability of mononuclear phagocytes to eradicate pathogens, and facilitates *M. tuberculosis* interaction with host (33, 34). Moreover, ESAT-6 is recognized as a potent T cell antigen, although it does not elicit a strong humoral immune response (4, 26, 27, 35, 36). Members of the PE/PPE protein family, such as PPE25 and PE19, play crucial roles in immunodominance and antigenic variation, contributing to mycobacterial virulence, persistence, and pathogenesis (37–40). Similarly, TB10.4 is a well-known secretory antigenic protein essential to mycobacterial pathophysiology, triggering a potent CD8^+^ T cell response (41, 42).

Emerging evidence suggests that, alongside T cell mediated cellular immunity, antibodies and B cells may offer protection against *M. tuberculosis* infection (9, 43–50). Antibodies targeting *M. tuberculosis* surface antigens can potentially mitigate the infection’s adverse effects by accelerating pathogen clearance and preventing pathogen entry into host cells. PE_PGRS33, a mycobacterial surface exposed antigen, interacts with host macrophage TLR2 to generate inflammatory signals and facilitate macrophage entry (51–54). The PGRS domain of PE_PGRS33 aids in the interaction with host TLR2, while the PE domain is necessary for protein translocation through the mycobacterial cell wall (55). Native recombinant PE_PGRS33-immunized mice were able to limit *M. tuberculosis* growth *in vivo* (51). Subjects vaccinated with BCG and those with latent tuberculosis infection (LTBI) produced antibodies against PE_PGRS33, primarily targeting epitopes in the PGRS domain (56). By neutralizing the interaction with TLR2, antibodies against PE_PGRS33 may disrupt a mycobacterial pathogenic pathway. Additionally, PE_PGRS33-specific antibodies may opsonize *M. tuberculosis*, facilitating more effective phagocytosis and destruction by activated macrophages (55). These findings highlight the potential of the PGRS domain of PE_PGRS33 as a target for a humoral immune response that neutralizes TB.

**Figure 1:**
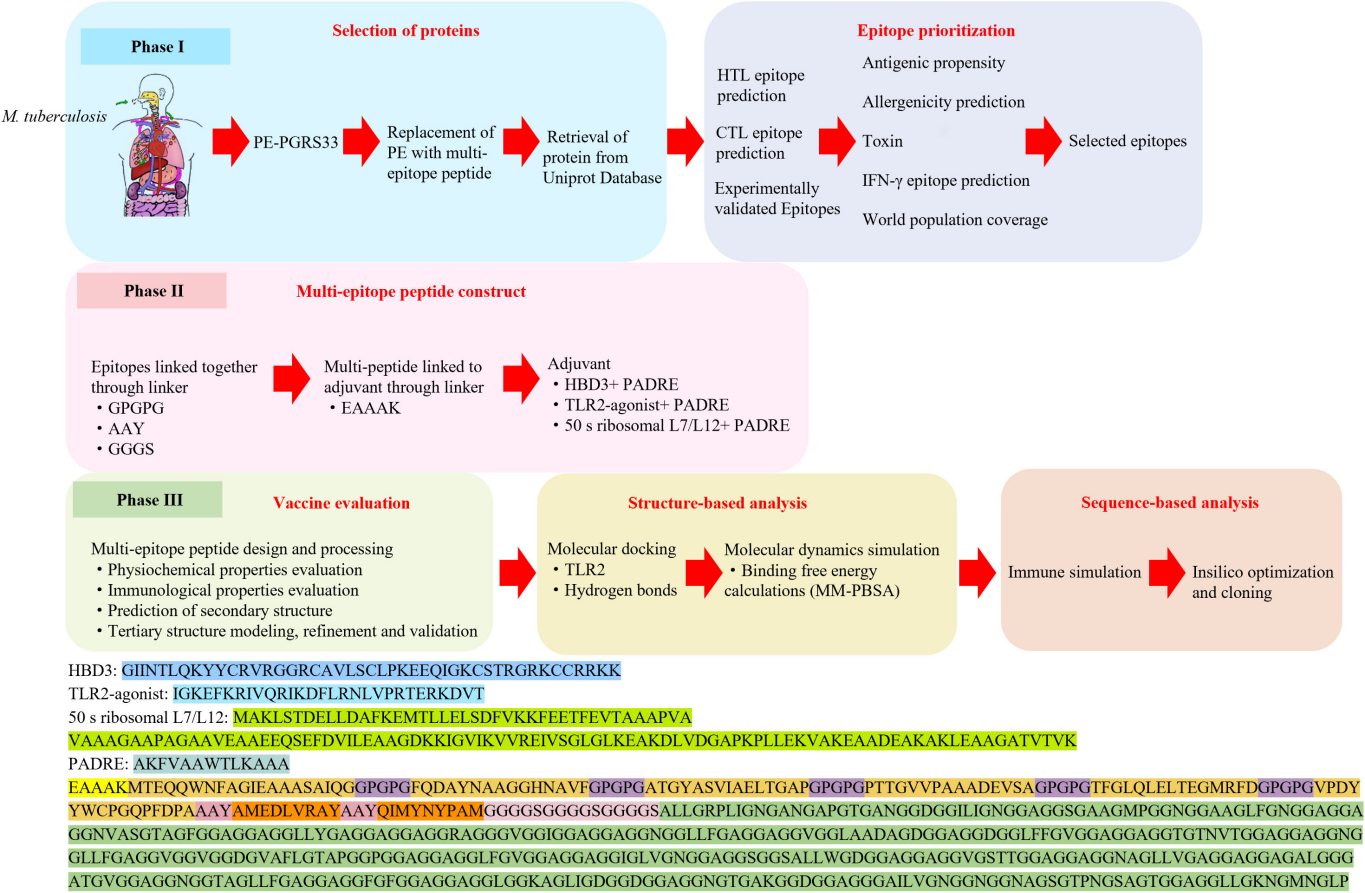
A schematic representation of the workflow used in developing a multi-epitope vaccine against *M. tuberculosis*.

Here, we adopted an epitope-based strategy focusing on adhesion-associated antigens that mediate the initial interaction of *M. tuberculosis* with host cells. Six helper T lymphocyte (HTL) epitopes were identified from ESAT-6, Ag85B, PPE25, PE19, HSP65, and MTP, together with two cytotoxic T lymphocyte (CTL) epitopes from TB10.4. These epitopes were combined with the PGRS domain of PE_PGRS33, which elicits strong humoral responses but lacks sufficient T cell epitopes. By assembling these elements into a rationally designed multi-epitope construct, we aimed to achieve a balanced stimulation of both cellular and humoral immunity (**Figure 1**).

The selection of epitopes was guided by both computational prediction and evidence from previous experimental studies. For ESAT-6 and Ag85B, we incorporated epitopes that have been consistently associated with robust CD4^+^ T cell responses (57, 58). PPE25 and PE19 epitopes were chosen based on a combination of in silico analysis and published experimental validation, ensuring reliable immunogenicity (59). Highly reactive epitopes from HSP65 and MTP were identified computationally, while two epitopes from TB10.4 were selected to cover both experimentally verified and predicted candidates (41). Appropriate linkers and adjuvant sequences were introduced to optimize antigen presentation and enhance vaccine potency.

To move beyond purely computational predictions, we further validated our design through wet-lab experiments. Among the three candidate constructs, the TLR2-agonist and PADRE-adjuvanted vaccine (TLR2-vaccine) was selected for *in vivo* testing. In mouse models, this vaccine not only reduced bacterial burden in the lung and other organs but also effectively prevented extrapulmonary dissemination. These results support the concept that targeting adhesins at the earliest stage of infection, combined with rational epitope design and optimized adjuvant selection, can provide protective immunity against *M. tuberculosis*.

## 2 Materials and methods

### 2.1 Sequence retrieval

Amino acid sequences of eight *Mycobacterium tuberculosis* (strain H37Rv) proteins—ESAT-6 (P9WNK7), Ag85B (P9WQP1), PPE25 (P9WI13), PE19 (Q79FK4), HSP65 (P9WPE7), MTP (P9WI87), TB10.4 (P9WNK3), and PE_PGRS33 (P9WIF5)—were retrieved from the UniProt database. The antigenicity of each protein was evaluated using the VaxiJen v2.0 and Immunomedicine Group servers (60, 61), while allergenicity and toxicity were assessed using AllerTOP v2.0 and ToxinPred, respectively. Only non-allergenic, non-toxic, and highly antigenic proteins were selected for further analysis (62–64).

### 2.2 Prediction of helper T lymphocyte epitope and IFN−γ inducing epitope

HTL epitopes were predicted using the IEDB MHC class II server with the human HLA-DR reference allele set. Fifteen-mer epitopes with the lowest percentile ranks were considered high-affinity candidates. The selected HTL epitopes were then screened for IFN-γ induction potential using the IFNepitope server based on Support Vector Machine algorithms (65–71).

### 2.3 Prediction of potential cytotoxic T lymphocyte epitope

CTL epitopes were predicted using the NetCTL 1.2 server, which integrates MHC class I binding, proteasomal cleavage, and TAP transport efficiency (72, 73). Epitopes with high combined scores and strong predicted binding affinities were selected for further analysis.

### 2.4 Prediction of population coverage

To estimate global immune coverage, the selected epitopes were analyzed using the IEDB Population Coverage tool under default parameters (74). This analysis evaluated the theoretical proportion of the human population that could potentially respond to the designed multi-epitope vaccine.

### 2.5 Construction of final vaccine

After identifying the optimal epitopes, three multi-epitope vaccine constructs were designed using distinct adjuvant combinations: (i) β-defensin 3 (HBD3) with PADRE, (ii) TLR2 agonist with PADRE, and (iii) 50 s ribosomal protein L7/L12 with PADRE. HBD3, an antimicrobial peptide involved in innate immunity, activates and polarizes dendritic cells, thereby bridging innate and adaptive immune responses (75–78). The TLR2 agonist enhances proinflammatory cytokine release and promotes antigen presentation through macrophages and DCs (79–83). L7/L12, a ribosomal protein with proven immunogenicity in subunit vaccines, was also evaluated as an alternative adjuvant (84–88). PADRE, a universal helper epitope with broad HLA-DR binding capacity, was incorporated to enhance CD4^+^ T cell activation and overall vaccine immunogenicity (83, 89–91).

To construct a stable and immunologically active chimeric vaccine, suitable linkers were applied between functional domains. The EAAAK linker was placed between adjuvants and epitopes to maintain structural rigidity and prevent steric interference (92, 93). GPGPG was used to connect HTL epitopes, facilitating MHC-II presentation and reducing junctional immunogenicity (87, 94). AAY linkers were applied between CTL epitopes to promote proteasomal processing and efficient recognition by cytotoxic T cells (87, 95). Finally, the GGGS linker connected the PGRS domain to the multi-epitope segment, providing flexibility for correct B-cell epitope folding and structural integrity (96).

### 2.6 Prediction of physicochemical properties and solubility of multi-epitope vaccine

The physicochemical characteristics of the vaccine construct, including the number of amino acids, molecular weight, theoretical isoelectric point (pI), instability index, aliphatic index, and grand average of hydropathicity (GRAVY), were analyzed using the ExPASy ProtParam tool (97). Protein solubility upon expression in *E. coli* was predicted using the SOLpro server, which employs a support vector machine–based approach to classify proteins as soluble or insoluble with associated probabilities.

### 2.7 Secondary structure prediction

The secondary structure of the designed vaccine constructs was predicted using PSIPRED and Prabi (GOR IV). PSIPRED predicts α-helices, β-strands, and coils based on PSI-BLAST outputs analyzed by neural networks, providing reliable accuracy for proteins lacking homologous structures (98). The GOR IV algorithm, implemented in the Prabi server, applies information theory and residue pair frequencies within a 17-residue window to determine secondary structure elements (99, 100). The combined use of these methods provided a consistent estimation of the proportion of helices, sheets, and coils, allowing evaluation of the structural stability and folding tendency of the multi-epitope vaccine.

### 2.8 Tertiary structure prediction, refinement and validation

The tertiary structure of the multi-epitope vaccine was predicted using the AlphaFold Server, which applies the AlphaFold 3 model for high-accuracy biomolecular structure prediction. The resulting model was refined using GalaxyRefine, which performs side-chain repacking and molecular dynamics–based relaxation to improve structural quality (101, 102).

Model validation was conducted through SAVES v6.1 (VERIFY 3D and PROCHECK modules) and ProSA-web (103–107). VERIFY 3D assessed sequence–structure compatibility, PROCHECK evaluated stereochemical geometry, and ProSA-web provided Z-score–based quality estimation. Together, these analyses confirmed the structural reliability of the vaccine model.

### 2.9 Molecular docking

Molecular docking is widely used to predict binding interactions between protein structures. The crystal structure of the human TLR2 complex (PDB ID: 6NIG) was obtained from the Protein Data Bank (https://www.rcsb.org) (108). The PDB file was visualized using PyMOL software (Version 4.6.0, SourceForge Headquarters, San Diego, USA), and non-TLR2 molecules were removed. The vaccine was docked with TLR2 using the ClusPro 2.0 server (109–112), HawkDock server (113–115), and GRAMM server (116). The binding affinities (ΔG) of the docked complexes were uniformly calculated using the PRODIGY server (117, 118), with the structure exhibiting the lowest score considered the optimal docking structure. Finally, the interactions within the docking complex were analyzed using the LigPlot+ software (Version 2.2, European Bioinformatics Institute, Cambridge, United Kingdom), which is based on JAVA.

### 2.10 Molecular dynamic simulation

Molecular dynamics (MD) simulations were employed to simulate the stability and dynamic parameters of the docked complex, thereby evaluating the stability of the vaccine construct. The best candidate docking complex underwent molecular dynamics simulations using GROMACS software (119). The detailed process involved generating the gro file of the complex system using the Amber14sb force field in GROMACS. Before the simulation, the docking complex was solvated in a cubic box of water molecules (SPC) and neutralized with appropriate ions. To alleviate initial spatial collisions, energy minimization of the complex was performed using the steepest descent method. Additionally, the entire system was equilibrated in NVT and NPT ensembles, with the system temperature raised to 300 K and the pressure maintained at 1 bar. A 100 ns molecular dynamics simulation was subsequently conducted. Post-simulation, molecular dynamics calculations were performed for parameters such as RMSD (root mean square deviation), RMSF (root mean square fluctuation), Rg (radius of gyration), and SASA (solvent-accessible surface area). Furthermore, to understand the overall motion of the complex, principal component analysis (PCA) was conducted using the GROMACS analysis tools, with projections related to the first two principal components (PC1 and PC2) being calculated. The overall flexibility of the docking complex was also defined and calculated using the online iMODS server (120). Molecular dynamics simulations were also performed using Discovery Studio. The molecular system was prepared by applying CHARMM force fields and solvated in a water box with neutralizing ions. After energy minimization to resolve steric clashes, the system was equilibrated under NVT and NPT ensembles. A production MD run was then conducted under controlled temperature and pressure conditions. Following the simulation, trajectory analysis was carried out to assess the structural stability and dynamic behavior of the system.

### 2.11 Host immune system simulation

The immune response profile of the designed vaccine was simulated using the C-ImmSim server (121–128). This in silico model reproduces the interactions between lymphoid (HTL, CTL, B cells, and plasma cells) and myeloid (macrophages and dendritic cells) lineages, enabling prediction of humoral and cellular immune responses. Simulation parameters were set at time intervals of 1, 84, and 168; simulation volume of 50; and 1000 steps with a random seed of 12345. The selected HLA alleles included A0101, A0201, B0702, B0801, DRB1_0101, and DRB1_1501. Vaccine injections were modeled without lipopolysaccharide (LPS), and the adjuvant level was fixed at 100.

### 2.12 Recombinant plasmid construction

The vaccine sequence was reverse-translated and optimized for expression in *E. coli* using the JCat server to achieve suitable GC content and a high codon adaptation index (CAI) (129, 130). BamHI and EcoRI restriction sites were added to the N- and C-termini, respectively, to facilitate cloning into the pET28a(+) vector. The recombinant construct was then modeled using SnapGene software.

### 2.13 Mycobacterium strains and mice

*Mycobacterium bovis* BCG and *M. tuberculosis* H37Ra strains were obtained from Zhifei Longcom Biopharmaceutical Co., Ltd. (Anhui, China) and maintained on Middlebrook 7H10 agar medium (Solarbio, Cat. No. LA7230). Specific pathogen-free (SPF) female C57BL/6 mice, aged 6 weeks, were purchased from the National Institutes for Food and Drug Control (Beijing, China). Mice were housed under pathogen-free conditions in the Animal Biosafety Level-2 (ABSL-2) facility at the Experimental Animal Center, Zhifei Lvzhu Biopharmaceutical Co., Ltd. (Beijing, China). All mice were fed a sterile commercial mouse diet and provided ad libitum access to water.

### 2.14 Mice immunization and bacterial challenge

TLR2-vaccine (10 μg/mouse) was dissolved in 100 μl PBS, emulsified with 100 μl incomplete Freund’s adjuvant (IFA), and administered subcutaneously three times at 2-week intervals. For BCG immunization, mice received 1 × 10^6^ CFU *M. bovis* BCG in 100 μl PBS via subcutaneous injection on the back.

For the preventive infection model, mice were challenged with 2 × 10^6^ CFU *M. tuberculosis* H37Ra in 100 μl PBS via tail vein. Two weeks later, lungs, livers, and spleens were harvested for bacterial load determination, and selected tissues were processed for hematoxylin and eosin (H&E) staining. Tissue homogenates were serially diluted 10-fold in PBS, and 100 μl of each dilution was plated on Middlebrook 7H10 agar (Solarbio, China). Plates were incubated at 37°C for 4 weeks before enumeration of colony-forming units (CFU). For the post-exposure protection model, mice were infected with aerosolized H37Ra using a Glas-Col inhalation exposure system (Terre Haute, IN, USA), adjusted to deliver an initial dose of ~200 CFU per mouse. Two weeks after infection, mice were immunized with TLR2-vaccine as described above, and bacterial loads and histopathology of the lungs and livers were assessed 2 weeks after the final immunization.

### 2.15 Serum antibody ELISA

Microtiter plates were coated with 100 µl of either TLR2-vaccine (2 µg/mL) or heat-inactivated H37Ra (1×10^5^ CFU/mL) in carbonate buffer (0.015 M Na_2_CO_3_/0.035 M NaHCO_3_, pH 9.6) and incubated overnight at 4°C. Plates were blocked with PBS containing 0.1% Tween-20 and 3% BSA at 37°C for 2 h, then washed with PBS/0.1% Tween-20. Mouse sera collected from the tail vein were diluted at an initial 1:100 and subjected to two-fold serial dilutions. One hundred microliters of each dilution were added to the wells and incubated at 37°C for 1 h. After washing, wells were incubated with HRP-conjugated anti-mouse IgG (1:5000; Sigma) in PBS/0.1% Tween-20/0.75% BSA for 1 h at room temperature. Plates were developed with TMB substrate for 5 min, stopped with 0.2 M H_2_SO_4_, and absorbance was read at 450 nm on a microplate reader. Endpoint titers were defined as the highest serum dilution giving an OD ≥ 2× the negative control.

### 2.17 IFN-γ ELISA (splenocyte stimulation and serum)

Single-cell suspensions of splenocytes were prepared and adjusted to 2 × 10^5^ cells per well for cytokine assays. Splenocytes were cultured in 96-well plates in complete RPMI medium and stimulated with antigen (20 µg/ml) for 72 h at 37°C with 5% CO_2_. Supernatants were harvested and stored at −80°C until analysis.

IFN-γ levels in both splenocyte culture supernatants and serum samples were measured using the ELISA MAX™ Standard Set Mouse IFN-γ (BioLegend, Cat. No. 430801), following the manufacturer’s instructions. All ELISA measurements were performed in duplicate.

### 2.18 Statistical analysis

All statistical analyses were performed using GraphPad Prism version 10.4.0 for Windows (GraphPad Software, La Jolla, CA, USA; www.graphpad.com). *P* values less than 0.05 were considered statistically significant. Statistical significance was indicated as follows: *p < 0.05, **p < 0.01, ***p < 0.001, ****p < 0.0001.

## 3 Results

### 3.1 Protein sequences, structures and immunological properties

In this study, we selected eight immunogenic proteins from *M. tuberculosis* (H37Rv strain): ESAT-6, Ag85B, PPE25, PE19, HSP65, MTP, TB10.4, and PE_PGRS33. These protein sequences were retrieved from the UniProt database, and their tertiary structures were obtained from the AlphaFold Protein Structure Database. For antigens with experimentally solved structures, such as ESAT-6–CFP10 complex (PDB ID: 1WA8) and the Ag85B (PDB ID: 1F0N), the corresponding PDB entries were also referenced. Because some available PDB structures are incomplete or represent complexes rather than full-length monomers, AlphaFold models were additionally used to visualize the complete conformations for epitope mapping (**Supplementary Figure S1**). These adhesins are associated with inhibiting the entry of *M. tuberculosis* into target cells. We performed immunogenicity predictions, epitope antigenicity evaluations, and physicochemical analyses to aid in vaccine development (**Supplementary Table S1**, **Supplementary Figure S2**). Using the AllerTOP v. 2.0 server, all proteins were confirmed to be non-allergenic (**Supplementary Table S1**). Toxicity assessments via the ToxinPred server showed that these proteins are non-toxic (**Supplementary Table S1**). Additionally, a non-homology search against the host proteome confirmed that all selected proteins are non-homologous.

### 3.2 Epitopes prediction

We utilized the IEDB server to predict epitopes for HTL. The HTL epitopes were chosen based on their top-ranking status, indicating their high affinity. ESAT-6, Ag85B, PPE25, PE19, HSP65, and MTP have been shown to trigger CD4^+^ T cell responses. We specifically selected epitopes from ESAT-6 and Ag85B that had previously demonstrated robust CD4^+^ T cell responses, while for the other four proteins, we predicted the HTL epitopes (**Supplementary Table S2**, **Supplementary Figure S3**).

Subsequently, we utilized the IFNepitope server to pinpoint HTL epitopes capable of inducing cell-mediated immunity. The antigenic regions that bind to MHC class II molecules and activate CD4^+^ T cells can stimulate IFN-γ production and initiate downstream signaling pathways. Each identified epitope was confirmed to enhance IFN-γ production (**Supplementary Table S2**).

For the CTL epitopes from TB10.4, we employed the NetCTL 1.2 server, which assessed several parameters critical for epitope efficacy. These included MHC-I binding affinity, rescaled MHC binding affinity, C-terminal cleavage affinity, transport efficiency, and a combined score, all summarized in **Supplementary Table S3**. MHC-I binding affinity is typically quantified as the half maximal inhibitory concentration (IC50) value. A lower IC50 value indicates a stronger binding affinity. The rescaled MHC binding affinity normalizes these values for uniform comparison. A high rescaled value indicates strong potential for T cell antigen presentation. The C-terminal cleavage affinity evaluates the likelihood of peptides being appropriately processed for MHC loading, where a balance is crucial to prevent over-degradation into suboptimal fragments. Transport efficiency measures the peptide’s ability to reach the endoplasmic reticulum for MHC loading. The combined score integrates these factors, providing a holistic assessment of each peptide’s potential as an epitope.

In designing a multi-epitope vaccine, we selected peptides with the best rating and those whose functionality has been experimentally validated. This approach ensures both the efficacy and reliability of the vaccine, targeting multiple critical epitopes.

### 3.3 Population coverage analysis

MHC molecules exhibit high polymorphism and are distributed extensively across different populations worldwide. This diversity underscores the potential of developing a broad-spectrum vaccine that effectively transcends ethnic boundaries. Utilizing a multi-epitope peptide-based vaccine approach is particularly advantageous under these circumstances.

To evaluate the potential population coverage, we used the IEDB analysis tool, which predicted the global coverage for both MHC class I and class II molecules based on the eight epitopes selected, as detailed in **Supplementary Figure S4** and **Supplementary Table S4**. The analysis revealed that the combined MHC class I and class II epitopes could potentially cover 98.55% and 99.99% of the global population, respectively. This extensive coverage suggests that these epitopes are promising candidates for the development of multi-epitope vaccines.

### 3.4 Construction of multi−epitope subunit vaccine

The final vaccine construct was assembled by integrating HTL and CTL epitopes along with the PGRS domain. Given the typically low immunogenicity of peptide vaccines, the inclusion of adjuvants is crucial to enhance their efficacy. In this research, we developed three distinct vaccine formulations, each featuring a different adjuvant combination: an HBD3 and PADRE vaccine (referred to as the HBD3-vaccine), a TLR2 agonist paired with PADRE (TLR2-vaccine), and a 50 s ribosomal protein L7/L12 with PADRE (50 s-vaccine). The structure of the vaccine included six HTL epitopes positioned adjacent to the adjuvant, followed by two CTL epitopes. Positioned at the end of the construct was the PGRS domain. These four domains were connected using specific linkers—EAAAK, GPGPG, AAY, and GGGS—as depicted in **Supplementary Figure S5**.

The helical EAAAK linker was strategically employed to connect the adjuvant to the epitopes, minimizing interactions with other protein regions while ensuring effective separation. The GPGPG linkers were chosen to enhance the immune response mediated by HTLs, and the AAY motif served as a linker to improve the separation of CTL epitopes, facilitating their efficient presentation. For connecting B cell epitope-enriched PGRS domain, the GGGS linker was used. This linker is known for its flexibility, which allows B cell epitopes to fold independently and function effectively while ensuring the overall structural stability of the vaccine construct.

### 3.5 Prediction of physiochemical properties, solubility, allergenicity and immunological properties of vaccine candidate

In this study, the final vaccine constructs were assessed for their physicochemical, solubility, allergenicity and immunological properties using ProtParam, SOLPro, AllerTOP v. 2.0 and VaxiJen 2.0 server (**Supplementary Table S5**).

The molecular weights of the three constructs were determined to be 53.6 kDa, 52.0 kDa, and 61.9 kDa, respectively. Typically, a molecular weight above 40 to 50 kDa facilitates lymphatic system uptake. All three vaccine candidates had molecular weights exceeding 50 kDa, indicating their suitability for effective lymphatic absorption (131). The stability of these constructs was gauged using the instability index; values below 40 suggest stability. The indices recorded were 23.28, 21.37, and 21.34, confirming the stable nature of our vaccines. Additionally, the aliphatic index, which reflects the volume occupied by aliphatic side chains and can influence protein thermostability, showed values of 60.77, 62.05, and 68.29, indicative of thermostability across varying temperatures (132). Hydropathy, assessed by the GRAVY, yielded values of 0.12, 0.137, and 0.183. These suggest a predominantly hydrophobic character of the vaccine proteins.

The solubility of the constructs, evaluated against a scaled solubility threshold (PopAvrSol) of 0.45, was also promising, with values of 0.969421, 0.992917, and 0.798146, demonstrating superior solubility compared to the average soluble protein from *E. coli*.

The allergenic potential of the vaccines was assessed using the AllerTOP 2.0 server, which confirmed their non-allergenic nature. Additionally, the antigenicity of these subunit vaccines was evaluated using the VaxiJen v2.0 server, with results of 1.6944, 1.6729, and 1.5185 against a threshold of 0.4, categorizing them as probable antigens. These assessments collectively underscore the high potential of the subunit vaccines in terms of stability, solubility, and antigenic capabilities.

### 3.6 Secondary and tertiary structures analysis

**Figure 2:**
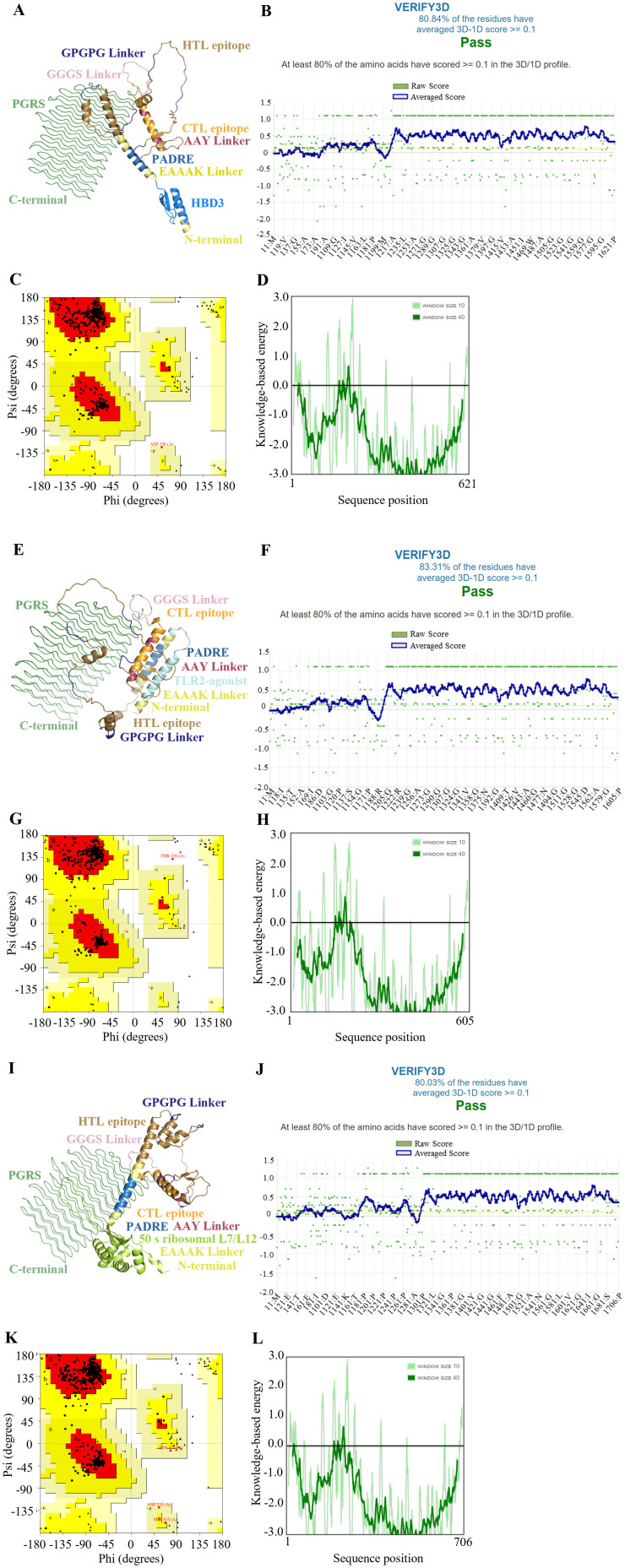
Structure characterization of multi-epitope vaccine. **(A-D)** HBD3-vaccine, **(E-H)** TLR2-vaccine, **(I-L)** 50 s-vaccine. **(A, E, I)** Tertiary structure, **(B, F, J)** The result of verification through the VERIFY3D tool, **(C, G, K)** The Ramachandran plot of the improved vaccine structure, **(D, H, L)** The energy profile of the vaccine candidate.

The secondary structures of the final vaccine constructs were analyzed using the Prabi server, with detailed results presented in **Supplementary Table S6**. The initial tertiary structures were generated by the AlphaFold Server, which provided an in-depth prediction of the 3D conformation of the vaccine constructs. These models were then refined using the GalaxyRefine web server to enhance their structural accuracy and reliability, as depicted in **Figures 2A, E, I** and **Supplementary Figure S6** offers a detailed evaluation score table for these refined models, which were crucial for selecting the most suitable refined models, as discussed in the subsequent analysis.

The quality of refinement was rigorously assessed using several metrics. The Global Distance Test - High Accuracy (GDT-HA) and RMSD quantified how closely the refined models mirrored the original structures, with values near 1 for GDT-HA and low RMSD values indicating a high degree of structural fidelity. The MolProbity score was utilized to evaluate the overall geometric quality of the models, where lower scores signify fewer geometrical errors and better structural integrity. Additionally, the clash score and poor rotamer score were examined to assess the number of steric clashes and the prevalence of unfavorable side-chain conformations, respectively, with lower scores denoting better outcomes. The Ramachandran plot favored percentage provides insight into the proportion of residues that adopt energetically favorable backbone conformations, with higher percentages reflecting better structural quality.

For further validation of the tertiary structure, we employed the SAVERS v6.1 and ProSA-web servers, corroborating our model’s structural integrity. Verification through the VERIFY3D tool, as shown in **Figures 2B, F, J**, confirmed that over 80% of residues in each model achieved scores above the threshold, indicative of well-folded and structurally compatible proteins. The Ramachandran plots (**Figures 2C, G, K**) confirmed that the majority of residues in all models were positioned within the most favored regions, which are energetically preferred.

The energy profiles of the vaccine candidates, evaluated using knowledge-based methods and illustrated in **Figures 2D, H, L**, generally showed negative values, suggesting stable intra-molecular interactions. Nonetheless, the observed fluctuations in the 50 s-vaccine model indicate areas where further optimization could enhance the vaccine’s stability and efficacy.

Overall, our analyses underscore the structural stability and potential efficacy of these vaccine candidates, with identified opportunities for refinement to further improve their effectiveness and stability.

### 3.7 Molecular docking analysis

Interactions between epitopes and immune cell receptors are critical for eliciting a sustained immune response from peptide vaccines. To evaluate the vaccine-receptor interactions, we performed molecular docking of the predicted optimal vaccine constructs with human TLR2. Multiple online tools, including ClusPro 2.0, HawkDock, and GRAMM, were utilized to increase the accuracy of docking predictions, each generating 10 docking results. To minimize discrepancies between different servers, the top three docking complexes from each server were evaluated for binding affinity (ΔG, kcal/mol) using the PRODIGY tool (**Supplementary Table S7**).

**Figure 3:**
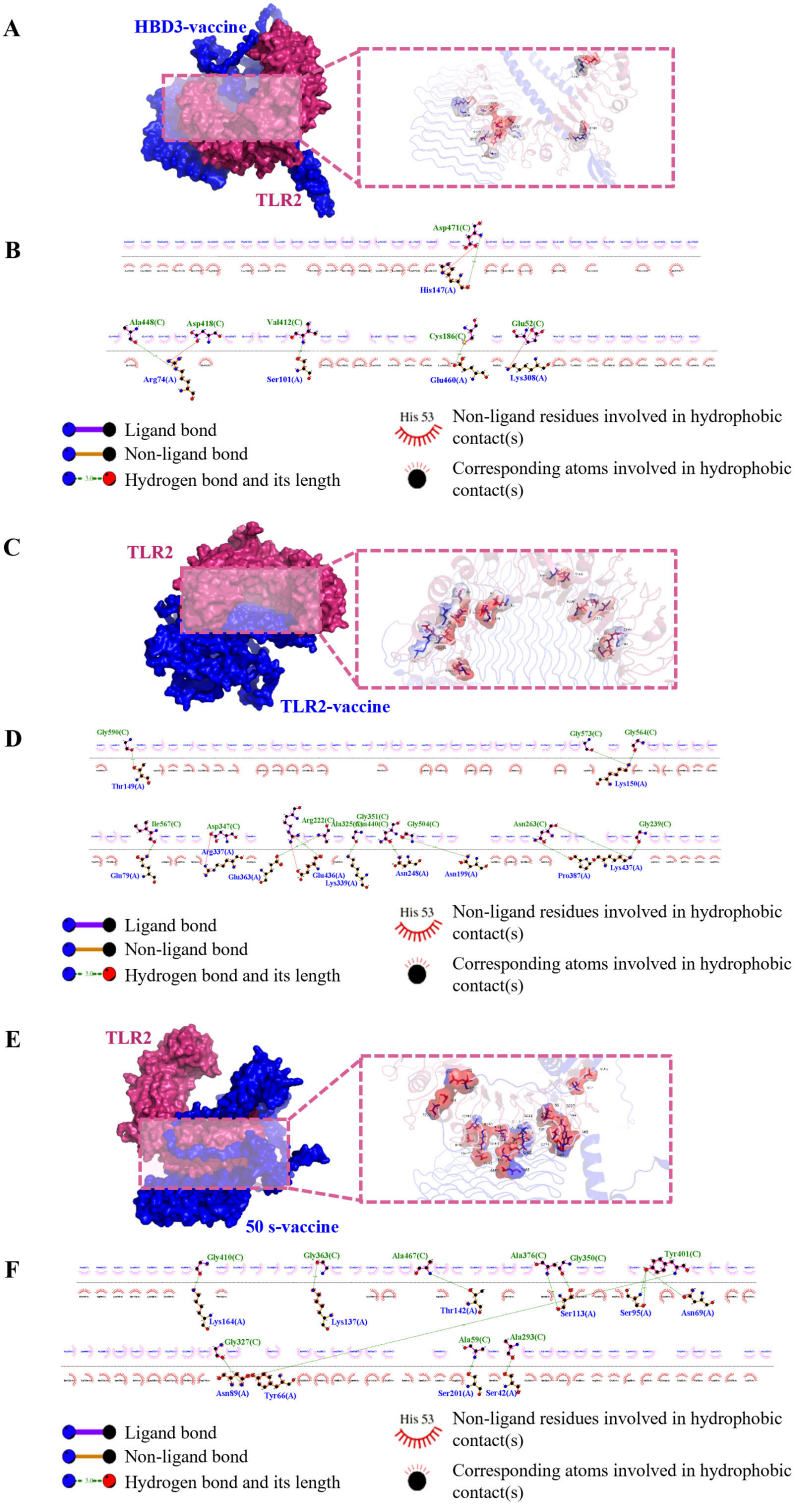
Visualization of molecular docking between vaccine candidate and TLR2. **(A, B)** HBD3-vaccine, **(C, D)** TLR2-vaccine, **(E, F)** 50 s-vaccine. **(A, C, E)** Results of molecular docking analysis for vaccine candidate with TLR2 predicted by GRAMM Server are shown. The left panel depicts a cartoon diagram of the molecular docking results, while the right panel displays a 3D zoomed-in diagram of the interactions between bonds at the molecular docking site. **(B, D, F)** 2D ligand-protein interaction diagram of the vaccine-TLR2 complex created using the LigPlot+ software.

The PRODIGY scoring results revealed that the docking complexes HBD3-vaccine-TLR2, TLR2-vaccine-TLR2, and 50 s-vaccine-TLR2 (GRAMM HBD3 model_3, GRAMM TLR2 model_2, and GRAMM 50 s model_1) exhibited the highest binding affinities, with ΔG values of −37.9 kcal/mol, −38.1 kcal/mol, and −49.5 kcal/mol, respectively. Visualization of these complexes was performed using PyMOL (**Figures 3A, C, E**), and 2D ligand-protein interaction diagrams were generated using LigPlot+ (**Figures 3B, D, F**). The HBD3-vaccine-TLR2 complex formed 7 hydrogen bonds, the TLR2-vaccine-TLR2 complex formed 14 hydrogen bonds, and the 50 s-vaccine-TLR2 complex formed 12 hydrogen bonds.

These results suggest that all three vaccine constructs can form stable interactions with TLR2, with the 50 s-vaccine-TLR2 complex demonstrating the highest binding affinity. Additionally, the TLR2-vaccine-TLR2 complex showed the highest number of hydrogen bonds, indicating potential stability. Given that hydrogen bonds are crucial for binding stability, the higher number of hydrogen bonds in the TLR2-vaccine-TLR2 complex may contribute to a strong and enduring immune response.

### 3.8 Molecular dynamics simulation study

Molecular dynamics (MD) simulations were employed to investigate the stability and conformational changes of vaccine constructs in complex with TLR2. Using GROMACS, 100 ns MD simulations were performed on the docked complexes, and their stability was assessed through RMSD, RMSF, and Rg metrics throughout the simulation period.

**Figure 4:**
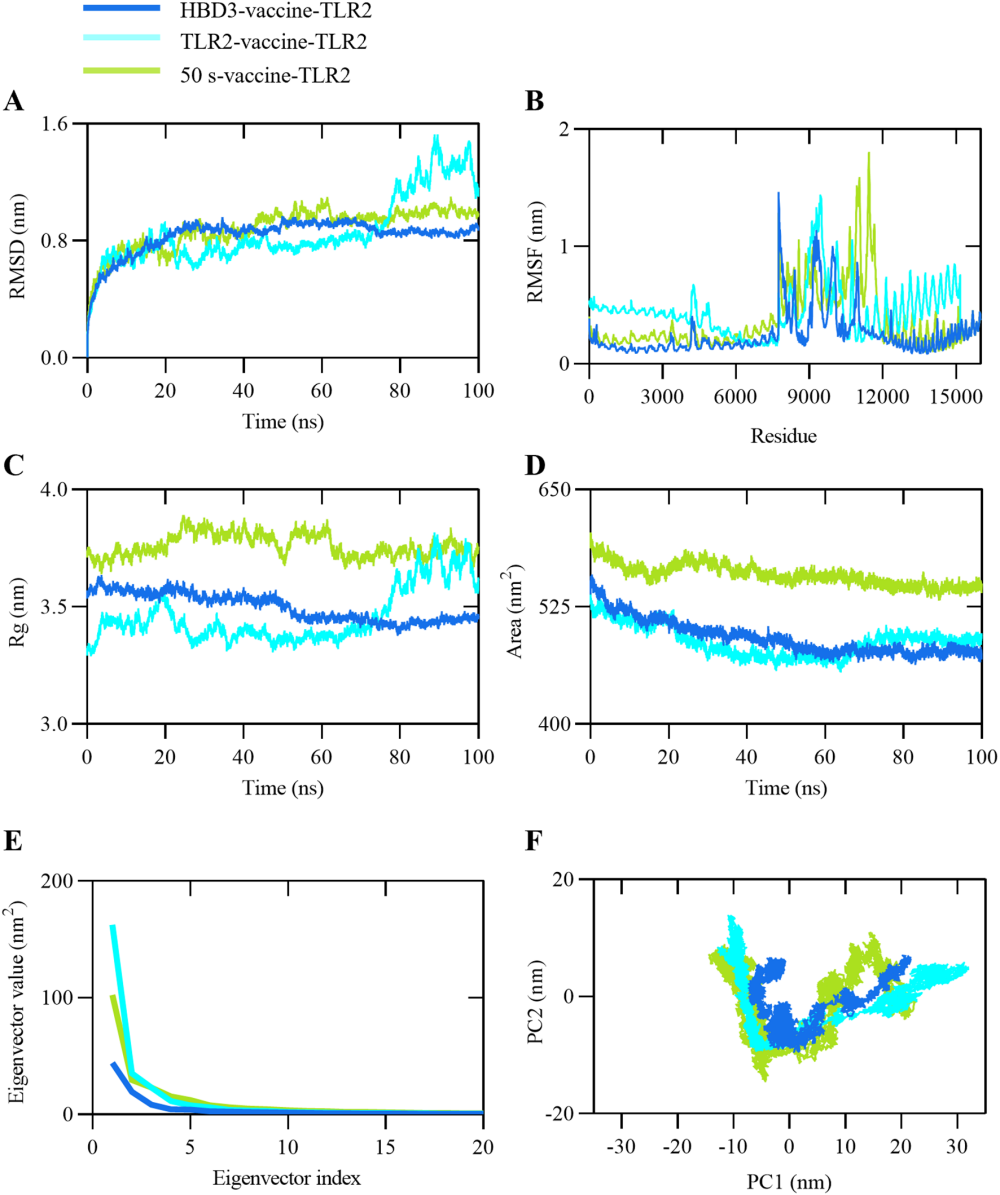
Representation of MD simulation analysis plot of proposed vaccine construct and TLR2 complex. **(A)** Root Mean Square Deviation (RMSD) of backbone atoms, **(B)** Root Mean Square Fluctuations (RMSF), **(C)** Radius of Gyration (Rg), **(D)** Solvent Accessible Surface Area (SASA) analysis, **(E)** Eigenvalues for the complex as a function of the first 20 eigenvectors, **(F)** 100 ns simulation trajectories projected onto the first two principal components (PCs), with the x-axis and y-axis representing PC1 and PC2, respectively.

RMSD provides insights into protein stability by measuring the variability of the protein structure. As shown in **Figure 4A**, HBD3-vaccine-TLR2 exhibited the lowest RMSD values throughout the simulation, indicating the highest structural stability. In contrast, TLR2-vaccine-TLR2 and 50 s-vaccine-TLR2 showed higher RMSD values in the later stages of the simulation, suggesting greater structural fluctuations and lower stability. RMSF was used to evaluate the flexibility and stability of residues within the complexes. **Figure 4B** demonstrates that HBD3-vaccine-TLR2 had the smallest residue fluctuations, further supporting its overall structural stability. Conversely, TLR2-vaccine-TLR2 and 50 s-vaccine-TLR2 exhibited larger residue fluctuations, particularly in specific regions, indicating higher flexibility in those areas.

Rg analysis was conducted to assess the compactness of the receptor-ligand complexes during the simulation. As depicted in **Figure 4C**, HBD3-vaccine-TLR2 had the lowest Rg values, indicating the most compact structure. 50 s-vaccine-TLR2 had the highest Rg values, suggesting a more loose structure, while TLR2-vaccine-TLR2 had Rg values in between the two. SASA was used to measure the surface area of the molecules exposed to the solvent. As shown in **Figure 4D**, both the HBD3-vaccine-TLR2 and the TLR2-vaccine-TLR2 exhibited similarly low SASA values, indicating a small surface area exposed to the solvent, which may correlate with improved stability. 50 s-vaccine-TLR2 had the highest SASA values, indicating the largest surface area exposed to the solvent and potentially lower stability. TLR2-vaccine-TLR2 had SASA values between the two.

Additionally, eigenvector analysis was performed to understand the overall motion and conformational changes of the docked complexes. The relationship between the first 20 eigenvalues and their respective indices was plotted in descending order (**Figure 4E**). The plot shows a rapid decrease in the magnitude of the first few eigenvalues, with HBD3-vaccine-TLR2 having the lowest eigenvector values, indicating the smallest structural changes. TLR2-vaccine-TLR2 and 50 s-vaccine-TLR2 had higher eigenvector values, indicating larger structural changes. The PCA scatter plot (PC1-PC2) was constructed by projecting the model trajectories onto two principal components, displaying the motion states of the complexes in a two-dimensional form (**Figure 4F**). The plot shows that HBD3-vaccine-TLR2 had the most concentrated motion pattern, indicating the smallest structural changes. TLR2-vaccine-TLR2 and 50 s-vaccine-TLR2 had more dispersed motion patterns, indicating larger structural changes.

In summary, the analysis indicates that HBD3-vaccine-TLR2 outperforms TLR2-vaccine-TLR2 and 50 s-vaccine-TLR2 in all aspects, demonstrating the highest structural stability and the most consistent dynamic behavior. TLR2-vaccine-TLR2 follows, while 50 s-vaccine-TLR2 exhibits the least stability and the most variable dynamic behavior.

MM-PBSA analysis was also employed to confirm the stability of the selected complexes throughout the molecular dynamics simulation. **Supplementary Table S8** presents the MM-PBSA values for the three complexes. Analyzing the energy components comprehensively, HBD3-vaccine exhibited the best performance in terms of Van der Waals energy, electrostatic energy, and gas-phase energy. Despite having a positive solvation energy, its total energy was negative, indicating the highest overall binding stability. Specifically, HBD3-vaccine had a Van der Waals energy of -236.53 kJ/mol, electrostatic energy of -1087.90 kJ/mol, and gas-phase energy of -1324.43 kJ/mol, with a solvation energy of 1266.63 kJ/mol, resulting in a total energy of -57.80 kJ/mol. TLR2-vaccine showed better performance in solvation energy compared to HBD3-vaccine but was inferior in other energy components, leading to a positive total energy and thus lower binding stability. TLR2-vaccine had a Van der Waals energy of -116.40 kJ/mol, electrostatic energy of 332.26 kJ/mol, gas-phase energy of 215.87 kJ/mol, and solvation energy of -198.08 kJ/mol, resulting in a total energy of 17.78 kJ/mol. 50 s-vaccine performed poorly across all energy components, particularly with positive values for both Van der Waals and electrostatic energies, resulting in the highest positive total energy and the least binding stability. 50 s-vaccine had a Van der Waals energy of 268.00 kJ/mol, electrostatic energy of 942.83 kJ/mol, gas-phase energy of 1210.83 kJ/mol, and solvation energy of -777.42 kJ/mol, resulting in a total energy of 433.40 kJ/mol. In summary, HBD3-vaccine demonstrated the highest binding stability, followed by TLR2-vaccine, while 50 s-vaccine exhibited the lowest binding stability.

**Figure 5:**
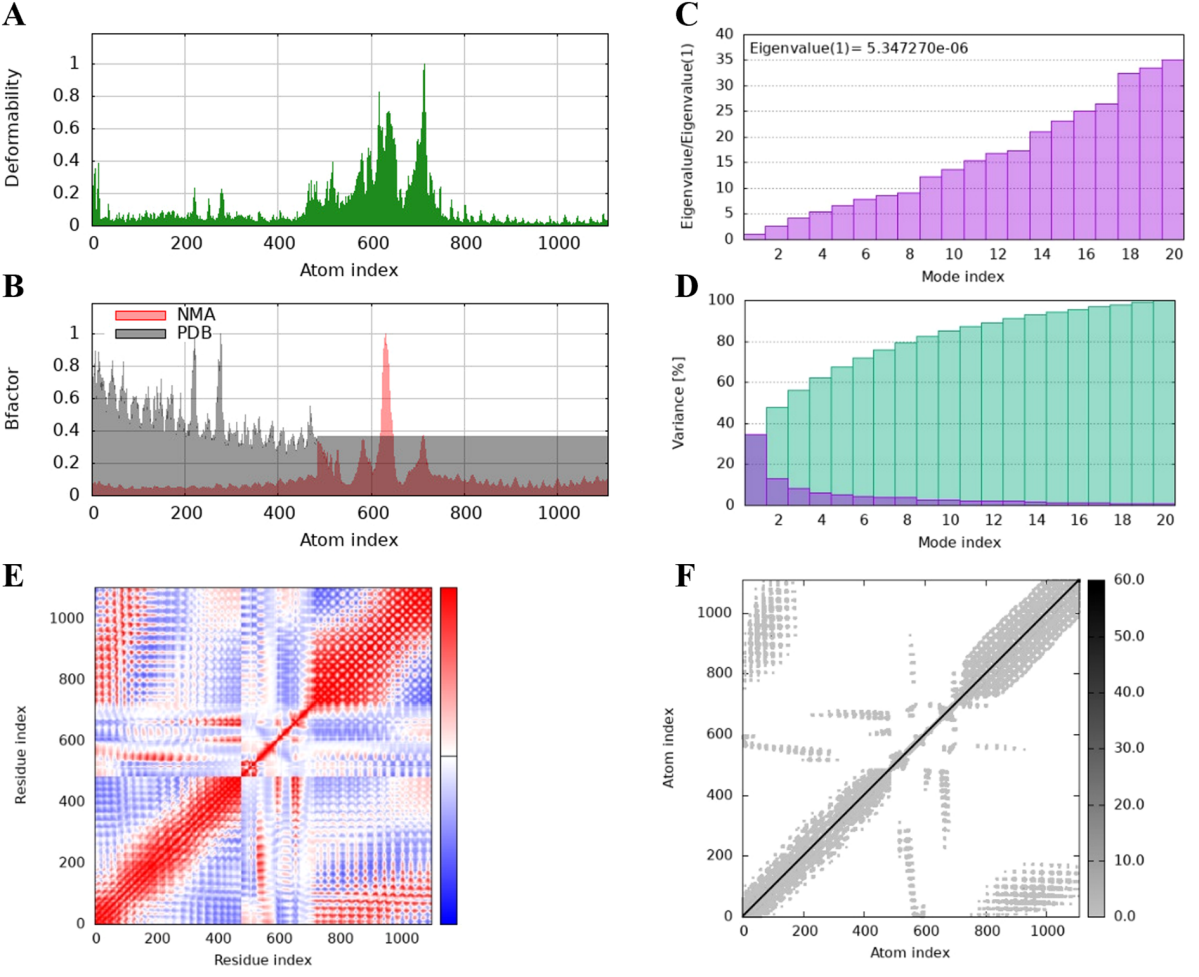
The results of molecular dynamics simulation of HBD3-vaccine and TLR-2 docked complex. **(A)** deformability, **(B)** B factor, **(C)** eigenvalues, **(D)** variance (purple color indicates individual variances and green color indicates cumulative variances), **(E)** co-variance map (correlated (red), uncorrelated (white) or anti-correlated (blue) motions) and **(F)** elastic network (darker gray regions indicate stiffer regions).

**Figure 6:**
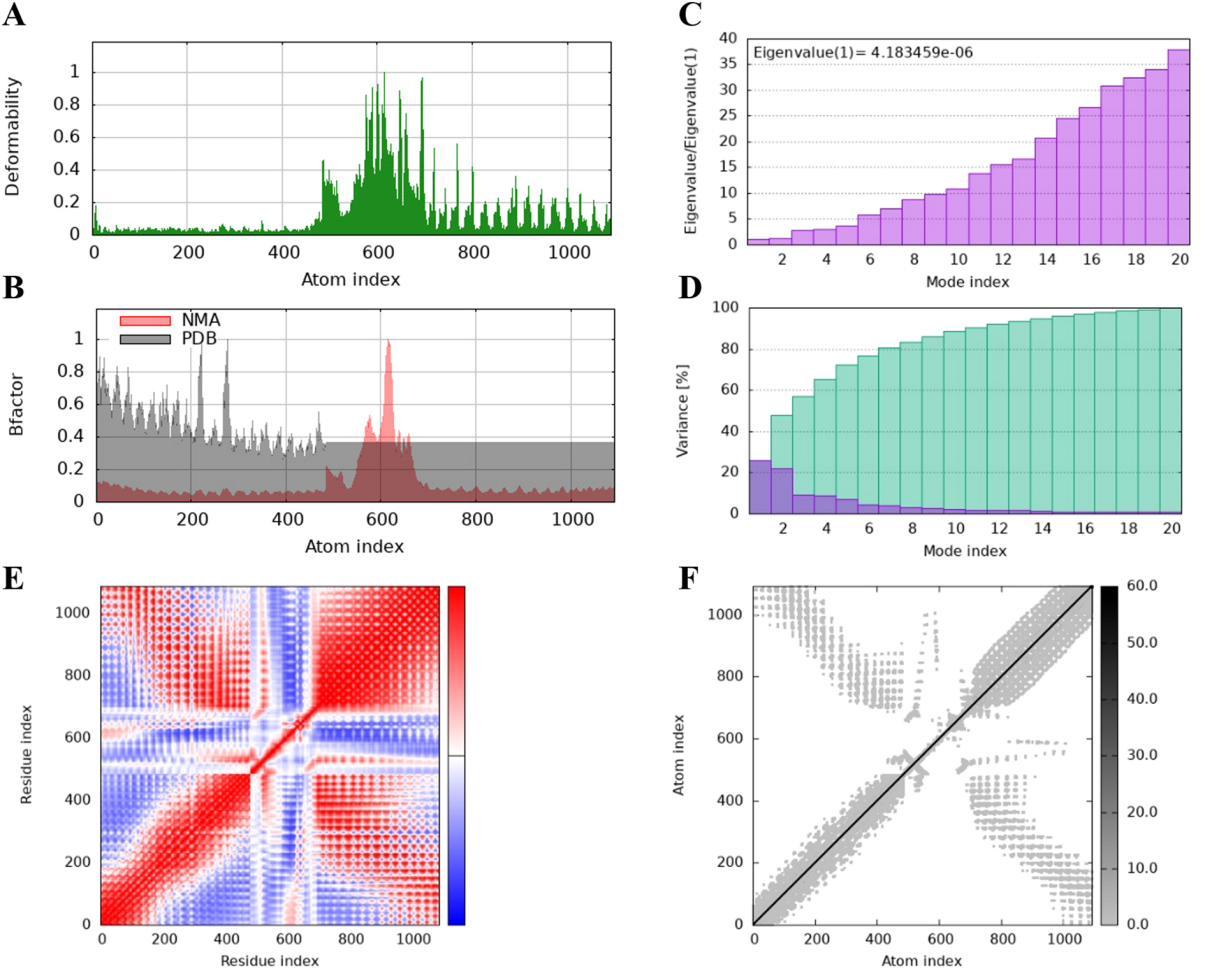
The results of molecular dynamics simulation of TLR2-vaccine and TLR2 docked complex. **(A)** deformability, **(B)** B factor, **(C)** eigenvalues, **(D)** variance (purple color indicates individual variances and green color indicates cumulative variances), **(E)** co-variance map (correlated (red), uncorrelated (white) or anti-correlated (blue) motions) and **(F)** elastic network (darker gray regions indicate stiffer regions).

**Figure 7:**
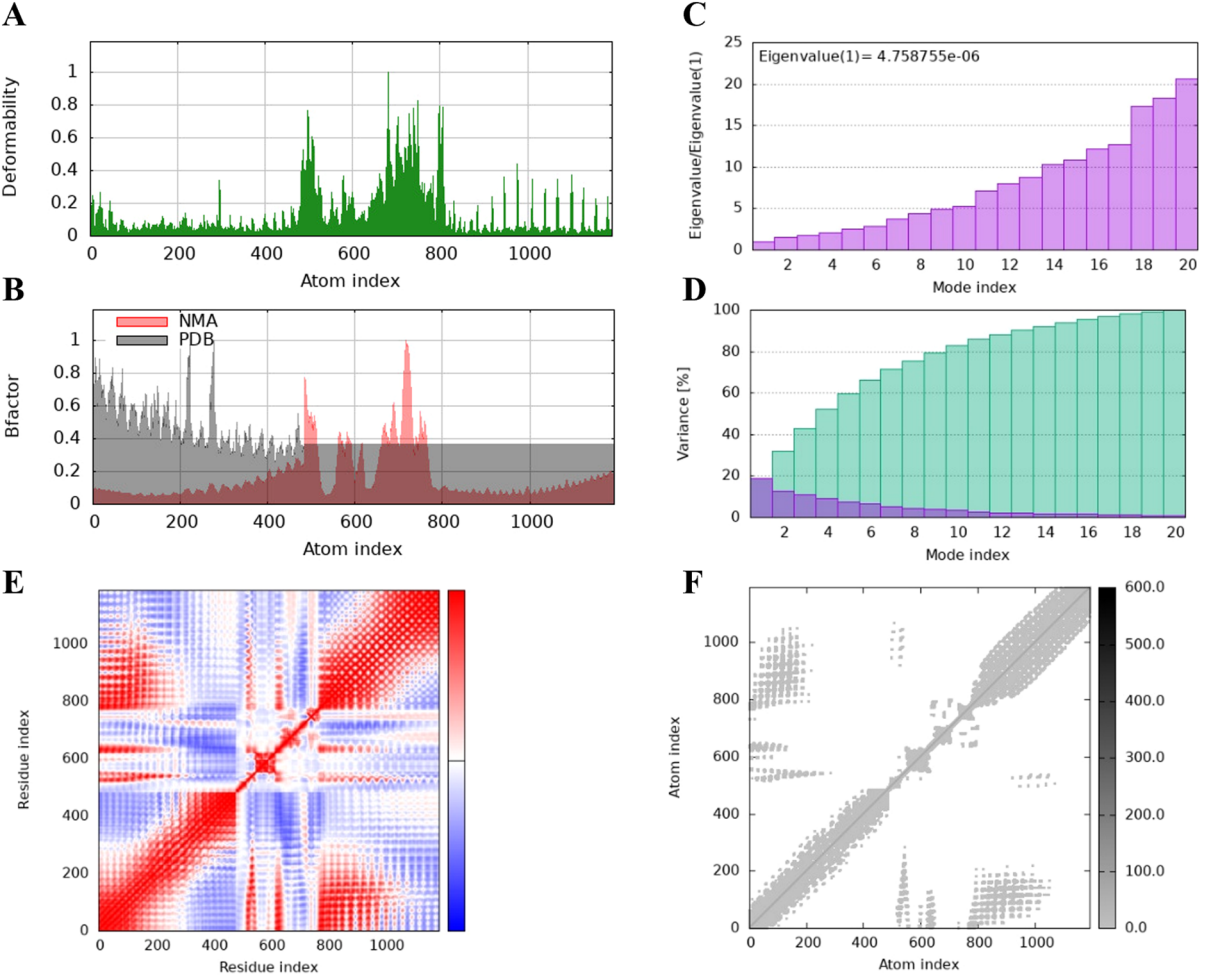
The results of molecular dynamics simulation of 50 s-vaccine and TLR2 docked complex. **(A)** deformability, **(B)** B factor, **(C)** eigenvalues, **(D)** variance (purple color indicates individual variances and green color indicates cumulative variances), **(E)** co-variance map (correlated (red), uncorrelated (white) or anti-correlated (blue) motions) and **(F)** elastic network (darker gray regions indicate stiffer regions).

The protein flexibility of the docking complexes was determined using the iMODs server, and the results were interpreted through deformability plots, B-factor plots, eigenvalues, variance plots, covariance maps, and elastic network models. The peaks in the deformability plots represent regions of the complex with higher flexibility, indicating areas that may need to adapt to TLR2 for effective docking (**Figures 5A**, **6A**, **7A**). Specifically, HBD3-vaccine-TLR2 and TLR2-vaccine-TLR2 showed prominent deformability in certain regions, suggesting localized adaptability during their interaction with TLR2. The B-factor plots were employed to assess the atomic mobility within the complex, as indicated by the relationship between the NMA (Normal Mode Analysis) and PDB (Protein Data Bank) sectors (**Figures 5B**, **6B**, **7B**). Among the three, TLR2-vaccine-TLR2 exhibited more extensive regions with higher B-factors, implying greater atomic flexibility and dynamic behavior during docking. The eigenvalues for the three complexes were 5.347270e-6, 4.183459e-6, and 4.758755e-6, respectively (**Figures 5C**, **6C**, **7C**). These values indicate the relative rigidity of each structure, with TLR2-vaccine-TLR2 showing the lowest eigenvalue, implying a more flexible overall conformation. In the variance plots, cumulative variance was represented in green, and individual variance in purple (**Figures 5D**, **6D**, **7D**). The eigenvalue and variance data revealed that each vaccine’s major dynamic modes contributed significantly to the overall motion, with TLR2-vaccine-TLR2 demonstrating higher cumulative eigenvalues and variance percentages in the initial modes. This suggests that the primary motions in TLR2-vaccine-TLR2 are more focused and potentially more stable.

The covariance maps depict the correlated (red), uncorrelated (white), and anti-correlated (blue) motions between residues (**Figures 5E**, **6E**, **7E**). The TLR2-vaccine-TLR2 complex showed a higher degree of positive correlation, indicating more coordinated dynamic behavior within the complex. Additionally, the elastic network models illustrated the density of elastic connections between atom pairs, where dark grey regions indicate more rigid areas of the structure (**Figures 5F**, **6F**, **7F**). The elastic network of TLR2-vaccine-TLR2 displayed more densely packed contact points, suggesting stronger binding stability compared to HBD3-vaccine-TLR2 and 50 s-vaccine-TLR2.

Additionally, Discovery Studio was used to visualize the docked complexes. Although the results were primarily visual and involved the generation of videos for the three docked complexes, the solvent was removed to enhance clarity (**Supplementary Videos 1**-**3**). This visualization provided a clearer understanding of the docking interactions and structural conformations of the complexes, albeit without the detailed quantitative analysis provided by other methods.

In summary, the molecular simulations performed using the iMODs server indicate that TLR2-vaccine-TLR2 exhibits superior performance across several metrics, including B-factors, eigenvalues, variance explanation, and contact maps. This suggests that TLR2-vaccine-TLR2 may form a more stable and coordinated docking complex with TLR2. While HBD3-vaccine-TLR2 and 50 s-vaccine-TLR2 display beneficial characteristics in certain areas, TLR2-vaccine-TLR2 demonstrates better overall stability and coordination, indicating its potential for more effective interaction with TLR2.

Combining the results from GROMACS analysis, MM-PBSA, and iMODs, HBD3-vaccine-TLR2 and TLR2-vaccine-TLR2 each show strengths in different aspects. However, overall, HBD3-vaccine-TLR2 demonstrates superior binding and structural stability. GROMACS analysis, including RMSD, RMSF, Rg, and SASA, indicates that HBD3-vaccine-TLR2 maintains a more stable structure during molecular dynamics simulations. MM-PBSA results further confirm the stability of HBD3-vaccine-TLR2, showing favorable van der Waals, electrostatic, and gas-phase energies, despite a positive solvation energy, resulting in a negative total energy indicating the most stable binding. Although iMODs results suggest that TLR2-vaccine-TLR2 performs well in terms of B-factors, eigenvalues, variance explanation, and contact maps, indicating potentially stable docking with TLR2, this finding is not entirely consistent with the GROMACS and MM-PBSA results. Notably, all three methods consistently indicate that 50 s-vaccine-TLR2 performs poorly in terms of energy components and structural stability, making it the least stable complex. Therefore, despite some support for TLR2-vaccine-TLR2 from iMODs results, the combined analysis from GROMACS and MM-PBSA suggests that HBD3-vaccine-TLR2 is the most optimal choice among the three vaccines due to its superior binding and structural stability.

### 3.9 Immune simulation

**Figure 8:**
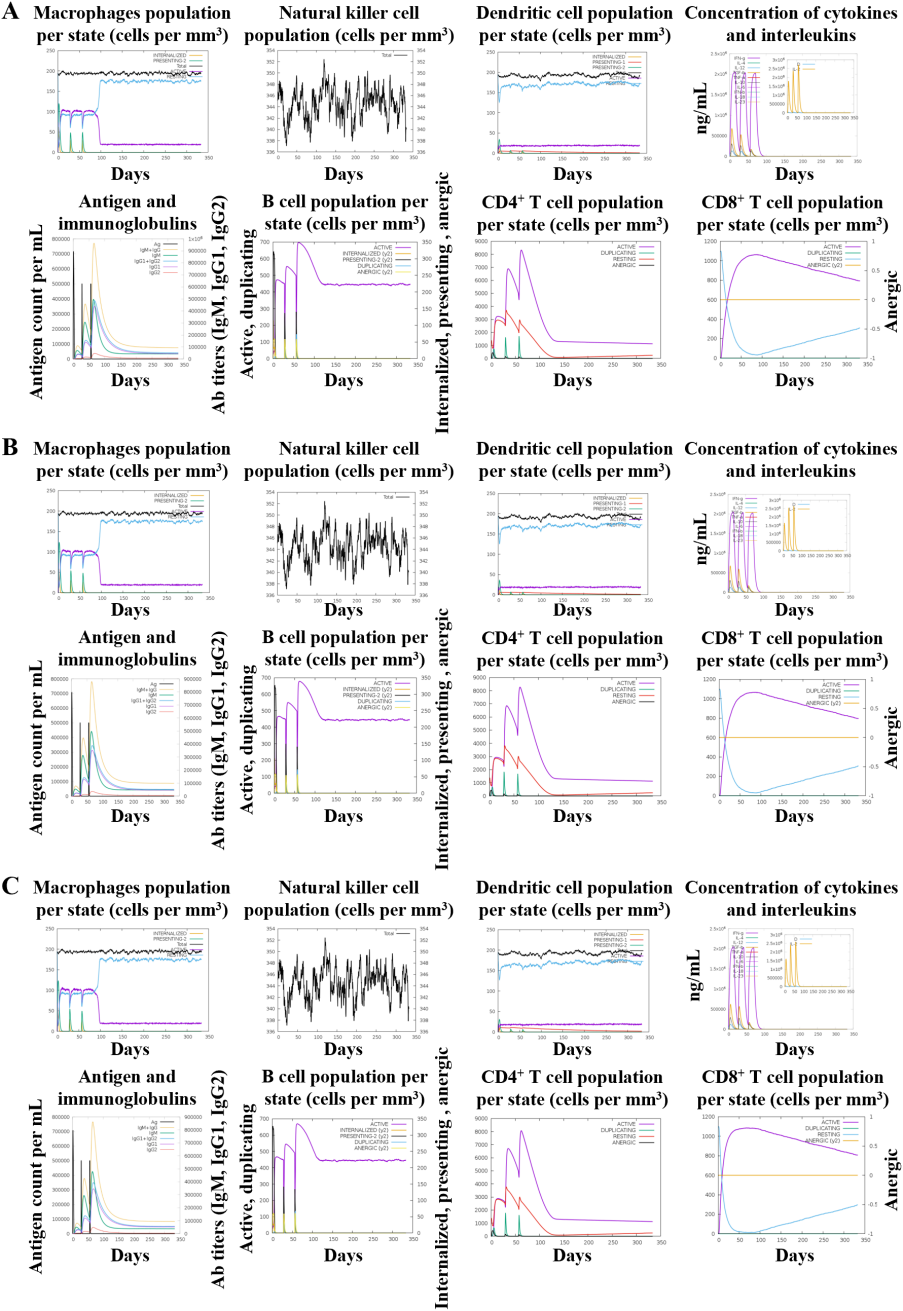
In silico immune simulation results of the vaccine construct using C-ImmSim are presented as follows: **(A)** HBD3-vaccine, **(B)** TLR2-vaccine, **(C)** 50 s-vaccine.

The C-ImmSim server was used to generate a preliminary in-silico prediction of innate and adaptive immune responses, providing theoretical guidance for vaccine evaluation (**Figure 8**). The HBD3-vaccine, TLR2-vaccine, and 50 s-vaccine showed stable innate immune responses with similar levels of macrophages, natural killer (NK) cells, and dendritic cells (DCs) across all vaccines. This indicated a controlled activation, preventing excessive inflammation. In adaptive immunity, B cell dynamics demonstrated a swift shift from IgM to more mature IgG antibodies, essential for a lasting immune defense. The activation patterns of both CD4^+^ and CD8^+^ T cells suggested effective helper and cytotoxic functions, critical for a strong immune response against pathogens.

Adaptive responses were further evidenced by robust cytokine production, including key mediators like IFN-γ, IL-2, and TNF-α, which were crucial for amplifying cellular immune responses. These cytokines facilitated the interaction between innate and adaptive immune components, reinforcing the body’s ability to mount a sustained defense.

Despite using the same antigenic epitopes, differences in adjuvant formulations between the HBD3-vaccine, TLR2-vaccine, and 50 s-vaccine influenced their efficacy. The HBD3 and TLR2 vaccines showed slightly enhanced immune responses, marked by higher cytokine levels and more vigorous B and T cell activations compared to the 50 s-vaccine. This suggests that the adjuvants in the HBD3 and TLR2 vaccines might boost the immune response more effectively, thus potentially offering better and more durable protection.

### 3.10 Codon optimization and cloning

The reverse translation and codon optimization of the final vaccine construct resulted in a CAI value of 1 for our optimized nucleotide sequence, indicating a high level of protein expression in *E. coli*. It is ideal to achieve a CAI value of 1, although values ranging from 0.8 to 1.0 are also considered acceptable. CAI values below 0.8 suggest poor expression of the target gene. Additionally, the GC content percentage plays a critical role in expression efficiency, with the optimal range being between 30% and 70% (14). Our three vaccine constructs exhibited GC content percentages of 66%, 67%, and 66%, respectively, indicating good expression efficiency in *E. coli* as they fall within the optimal range.

To facilitate the cloning process of the vaccine construct into *E. coli* pET-28a(+) vectors, we introduced BamHI and EcoRI restriction sites at the N-terminal and C-terminal ends of the optimized codon sequence, respectively. Subsequently, the optimized codons with restriction sites were inserted into the vector through restriction cloning. This process resulted in the generation of three cloned constructs with sequence lengths of 7232, 7184, and 7487 base pairs (**Supplementary Figure S7**).

Based on a comprehensive computational assessment—including molecular docking, dynamics simulations, and immune simulations—the TLR2-vaccine construct was prioritized for experimental validation. While all three candidates exhibited favorable in silico properties, the TLR2-vaccine demonstrated the most balanced profile, combining stable binding with TLR2, favorable energetic features, and conformational flexibility. Importantly, its immune simulation results suggested robust activation of both innate and adaptive responses, supporting its potential to elicit protective immunity. The optimized gene sequence of the TLR2-vaccine was synthesized, cloned into the pET-28a(+) vector, expressed in E. coli, and purified as a recombinant protein. This provided sufficient material for subsequent *in vitro* and *in vivo* immunogenicity evaluation.

### 3.11 Immunogenicity and protective efficacy of the TLR2-vaccine

**Figure 9:**
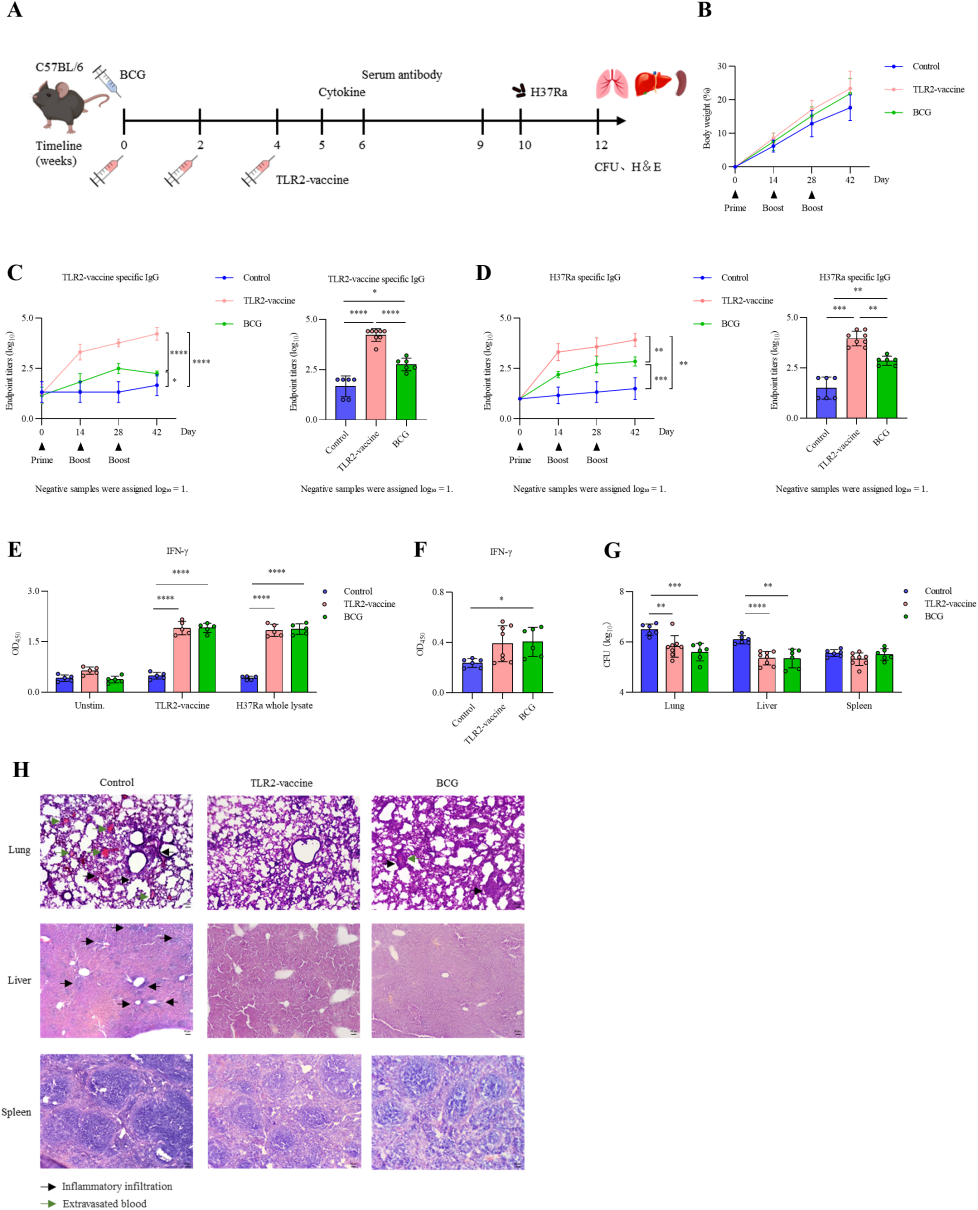
Immunogenicity and protective efficacy of TLR2-vaccine in C57BL/6 mice. **(A)** Experimental design. C57BL/6 mice (*n* = 12–14 per group) were immunized three times with PBS, BCG, or TLR2-vaccine. At week 5, six mice per group were sacrificed for splenocyte assays, and the remaining 6–8 mice per group were challenged with *M. tuberculosis* H37Ra via tail vein injection. **(B)** Body weight changes during immunization. **(C, D)** Serum antigen-specific IgG levels. **(C)** TLR2-vaccine–specific IgG; **(D)** H37Ra-specific IgG. Left, longitudinal antibody kinetics; right, endpoint titers two weeks after the final boost (statistical analysis was performed on the endpoint titers at the final time point). **(E)** Splenocyte IFN-γ production at week 5. **(F)** Serum IFN-γ levels two weeks post-challenge. **(G)** Bacterial loads in lungs, livers, and spleens after challenge. **(H)** Representative H&E staining of lungs, livers, and spleens. Control mice displayed severe inflammation, whereas TLR2-vaccine–immunized mice preserved tissue structure with only mild infiltration. Data are representative of three independent experiments, each with six mice per group. Statistical analyses were performed using one-way ANOVA followed by Tukey’s multiple comparisons test. *P<0.05; **P < 0.01; ***P < 0.001; **P < 0.0001.

Mice were immunized according to the schedule shown in **Figure 9A**. A subset of mice was sacrificed at week 5 for splenocyte assays, while the remaining mice were challenged with *M. tuberculosis* H37Ra at week 10. Body weight monitoring revealed no significant differences among control, BCG, and TLR2-vaccine groups, indicating good tolerability (**Figure 9B**). The TLR2-vaccine induced robust antigen-specific IgG responses, with antibody levels rising progressively after each boost and reaching significantly higher endpoint titers than the BCG group (**Figure 9C**). The modest antibody increase observed in the BCG group may reflect cross-reactivity with epitopes conserved across mycobacteria, as the TLR2-vaccine epitopes are derived from *M. tuberculosis* proteins. Similar results were obtained when H37Ra lysate was used as the coating antigen (**Figure 9D**). Splenocyte restimulation assays further showed that both the TLR2-vaccine and BCG groups secreted elevated levels of IFN-γ upon exposure to vaccine antigen or H37Ra lysate compared with the control group, confirming effective induction of Th1-type cellular responses (**Figure 9E**).

Protective efficacy was next evaluated following intravenous challenge with H37Ra. At two weeks post-infection, serum IFN-γ levels were higher in the TLR2-vaccine and BCG groups than in the control group, although variability was greater in the TLR2-vaccine group (**Figure 9F**). Bacterial load analysis demonstrated that both vaccines reduced colony counts in the lungs and liver, while no clear difference was observed in the spleen (**Figure 9G**). Histopathological examination supported these findings: control mice displayed extensive lung inflammation, alveolar collapse, and hemorrhage, whereas lungs from the TLR2-vaccine group largely maintained alveolar structure with only mild, scattered inflammatory cell infiltration. The BCG group showed intermediate pathology, with small granuloma-like aggregates and focal hemorrhage (**Figure 9H**). Liver inflammation was also alleviated in the TLR2-vaccine and BCG groups, while spleen morphology remained comparable across groups. Together, these results demonstrate that the TLR2-vaccine elicits both humoral and cellular immunity and confers protection against *M. tuberculosis* H37Ra comparable to BCG.

### 3.12 TLR2-vaccine limited extrapulmonary dissemination following pulmonary *M. tuberculosis* infection

**Figure 10:**
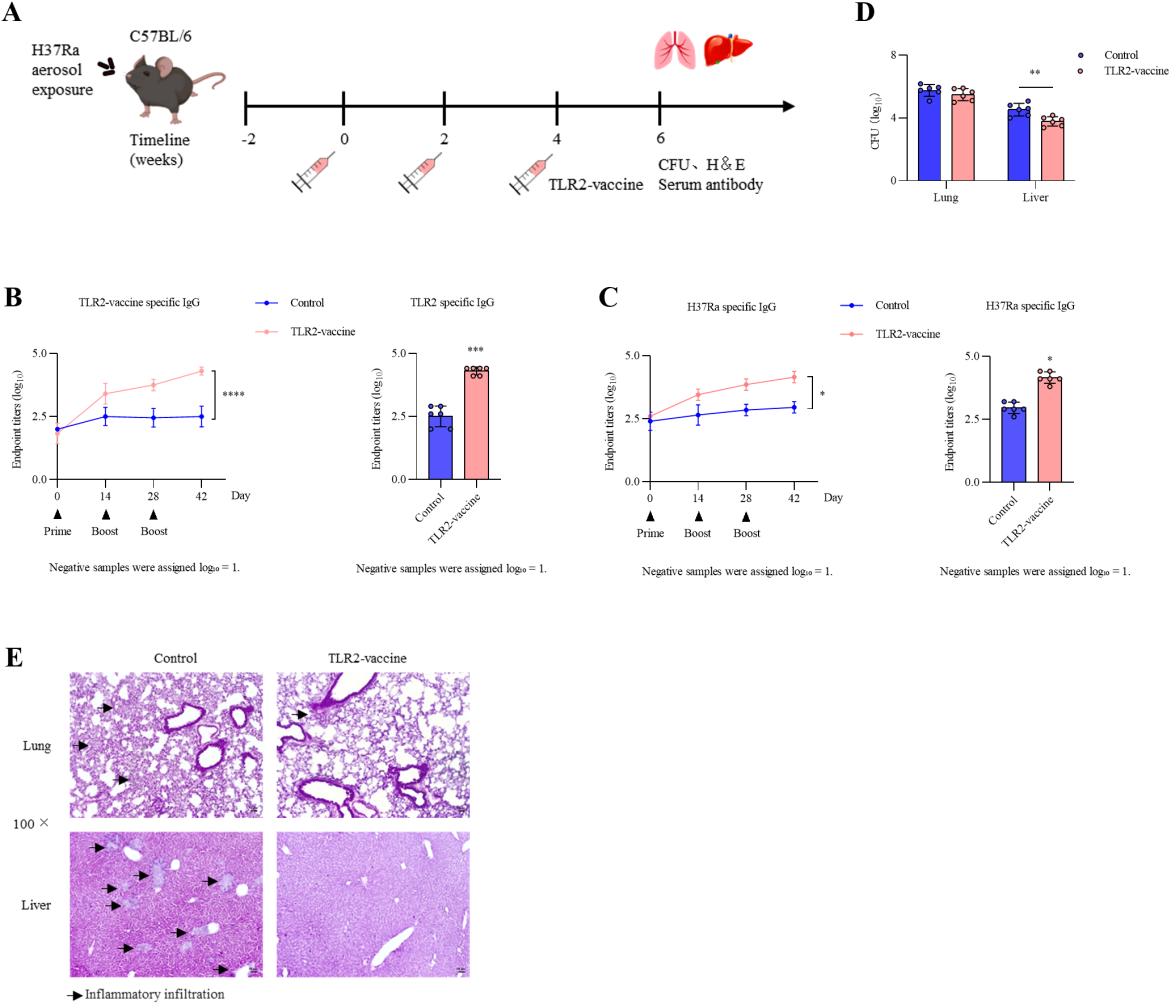
Post-infection immunization with TLR2-vaccine. **(A)** Experimental design. C57BL/6 mice (*n* = 6 per group) were challenged with aerosolized H37Ra. Two weeks later, mice were immunized three times at 2-week intervals with TLR2-vaccine, while the control group received no vaccine. All mice were sacrificed two weeks after the final immunization. **(B, C)** Antibody responses against TLR2-vaccine **(B)** and H37Ra **(C)**. Left: dynamic IgG titers during immunization; Right: endpoint titers two weeks after the final boost (statistical analysis was performed on the endpoint titers at the final time point). **(D)** Bacterial loads in lung and liver. **(E)** Representative H&E staining of lung and liver tissues. Data are representative of three independent experiments (*n* = 6 per group each) and analyzed using unpaired t-tests. *P<0.05; **P < 0.01; ***P < 0.001; **P < 0.0001; ns, not significant.

To assess whether the TLR2-vaccine could restrict bacterial dissemination after pulmonary infection, mice were first challenged with aerosolized *M. tuberculosis* H37Ra and subsequently immunized three times at 2-week intervals (**Figure 10A**). Antibody analysis showed that both TLR2-vaccine–specific IgG and H37Ra-specific IgG increased over the course of immunization (**Figures 10B, C**). Although control mice exhibited modest antibody induction following infection, vaccination elicited markedly higher titers, as confirmed two weeks after the final boost.

Bacterial burden analysis revealed no significant differences in the lung between groups; however, liver bacterial loads were significantly reduced in the TLR2-vaccine group compared with controls (**Figure 10D**). We focused on lung and liver as representative organs, given that the lung is the primary site of infection and the liver is a frequent extrapulmonary target during dissemination, whereas the spleen primarily functions as a secondary lymphoid organ and was not the focus of dissemination analysis. Histopathological examination further supported these findings: control mice showed focal inflammatory infiltrates in the liver and extensive pulmonary inflammation, whereas vaccinated mice exhibited preserved liver architecture and less severe lung inflammation (**Figure 10E**). These findings indicate that post-infection immunization with the TLR2-vaccine can enhance antigen-specific immune responses and restrict extrapulmonary bacterial dissemination.

## 4 Discussion

This study combined reverse vaccinology and experimental immunology to develop a multi-epitope subunit vaccine targeting *M. tuberculosis* adhesins. By integrating in silico analyses with *in vivo* validation, we demonstrated that the TLR2-vaccine induced strong humoral and cellular immune responses and effectively limited bacterial burden and dissemination in mice.

The correlation between bioinformatics predictions and experimental outcomes supports the reliability of computational vaccine design. The epitopes predicted to be highly antigenic and IFN-γ–inducing in silico indeed elicited robust Th1-type responses and high IgG titers *in vivo*. Similarly, molecular docking and immune simulations predicted stable binding between the vaccine construct and TLR2, suggesting a potential role of TLR2 engagement in the observed immune activation. These findings validate the predictive accuracy of our computational approach and highlight the feasibility of integrating in silico screening with experimental verification to accelerate TB vaccine development.

One limitation of this study is that we did not examine the sequence homology of the selected antigens with non-tuberculous mycobacteria (NTM). Previous reports suggest that several of these antigens, including ESAT-6 and Ag85B, have homologs in certain NTM species, though the degree of conservation and expression varies among strains (133–136). This potential overlap could influence immune recognition in populations frequently exposed to NTM, warranting further comparative analysis in future studies.

The protective mechanisms of the TLR2-vaccine likely involve the synergistic activation of both innate and adaptive immunity. The engagement of TLR2 by the vaccine construct may promote antigen presentation and IFN-γ–mediated Th1 responses, which are crucial for controlling intracellular *M. tuberculosis*. Additionally, the presence of B-cell epitopes from the PGRS component could facilitate antibody-mediated neutralization and opsonization, contributing to bacterial clearance. However, these mechanisms were not directly examined in this study and will require detailed immunophenotyping and cytokine analyses in future experiments.

Another aspect not addressed here is the evaluation of a prime–boost strategy with BCG. Given that BCG remains the foundation of TB vaccination programs, assessing the TLR2-vaccine as a booster following BCG priming could better reflect its translational potential. Future studies will explore this approach, along with testing against virulent clinical strains and assessing long-term immune memory.

In summary, our study demonstrates that the integration of computational prediction and experimental validation provides a powerful framework for TB vaccine discovery. The TLR2-vaccine showed consistent performance across in silico and *in vivo* analyses, eliciting broad and balanced immune protection. Further investigations into its immunological mechanisms, interaction with BCG priming, and potential cross-reactivity with NTM will help refine its development toward clinical application.

## Data Availability

The original contributions presented in the study are included in the article/**Supplementary Material**. Further inquiries can be directed to the corresponding author/s.

## References

[1] Adam T Baddeley A Bastard M Den Boon S Dean A . WHO's global tuberculosis report 2023. World Health Organ. (2023).

[2] Alsayed SSR Gunosewoyo H . Tuberculosis: pathogenesis, current treatment regimens and new drug targets. Int J Mol Sci. (2023) 24:5202. doi: 10.3390/ijms24065202, PMID: 36982277 PMC10049048

[3] Tait DR Hatherill M Van Der Meeren O Ginsberg AM Van Brakel E Salaun B . Final analysis of a trial of M72/AS01(E) vaccine to prevent tuberculosis. N Engl J Med. (2019) 381:2429–39. doi: 10.1056/NEJMoa1909953, PMID: 31661198

[4] Tkachuk AP Bykonia EN Popova LI Kleymenov DA Semashko MA Chulanov VP . Safety and immunogenicity of the GamTBvac, the recombinant subunit tuberculosis vaccine candidate: A phase II, multi-center, double-blind, randomized, placebo-controlled study. Vaccines (Basel). (2020) 8:652. doi: 10.3390/vaccines8040652, PMID: 33153191 PMC7712213

[5] Choi YH Kang YA Park KJ Choi JC Cho KG Ko DY . Safety and immunogenicity of the ID93 + GLA-SE tuberculosis vaccine in BCG-vaccinated healthy adults: A randomized, double-blind, placebo-controlled phase 2 trial. Infect Dis Ther. (2023) 12:1605–24. doi: 10.1007/s40121-023-00806-0, PMID: 37166567 PMC10173211

[6] Vidal SJ Lasrado N Tostanoski LH Chaudhari J Mbiwan ER Neka GD . Mining the CD4 antigen repertoire for next-generation tuberculosis vaccines. Cell. (2025) 15:S0092-8674(25)00982-1. doi: 10.1016/j.cell.2025.08.027, PMID: 40957415 PMC12445596

[7] Dagnew AF Han LL Naidoo K Fairlie L Innes JC Middelkoop K . Safety and immunogenicity of investigational tuberculosis vaccine M72/AS01(E-4) in people living with HIV in South Africa: an observer-blinded, randomised, controlled, phase 2 trial. Lancet HIV. (2025) 12:e546–55. doi: 10.1016/s2352-3018(25)00124-9, PMID: 40614747 PMC12310912

[8] Zhou S Cao Q Zhang Z Du Y Hou Y Zhang X . The adjuvant effect of manganese on tuberculosis subunit vaccine Bfrb-GrpE. NPJ Vaccines. (2024) 9:248. doi: 10.1038/s41541-024-01049-x, PMID: 39702587 PMC11659584

[9] Yang H Lei X Chai S Su G Du L . From pathogenesis to antigens: the key to shaping the future of TB vaccines. Front Immunol. (2024) 15:1440935. doi: 10.3389/fimmu.2024.1440935, PMID: 39108269 PMC11300335

[10] Li W Joshi MD Singhania S Ramsey KH Murthy AK . Peptide vaccine: progress and challenges. Vaccines (Basel). (2014) 2:515–36. doi: 10.3390/vaccines2030515, PMID: 26344743 PMC4494216

[11] Goodswen SJ Kennedy PJ Ellis JT . A guide to current methodology and usage of reverse vaccinology towards in silico vaccine discovery. FEMS Microbiol Rev. (2023) 47:fuad004. doi: 10.1093/femsre/fuad004, PMID: 36806618

[12] Jacob CO Leitner M Zamir A Salomon D Arnon R . Priming immunization against cholera toxin and E. coli heat-labile toxin by a cholera toxin short peptide-beta-galactosidase hybrid synthesized in E. coli. EMBO J. (1985) 4:3339–43. doi: 10.1002/j.1460-2075.1985.tb04086.x, PMID: 3004953 PMC554663

[13] Hajighahramani N Eslami M Negahdaripour M Ghoshoon MB Dehshahri A Erfani N . Computational design of a chimeric epitope-based vaccine to protect against Staphylococcus aureus infections. Mol Cell Probes. (2019) 46:101414. doi: 10.1016/j.mcp.2019.06.004, PMID: 31233779

[14] Ali M Pandey RK Khatoon N Narula A Mishra A Prajapati VK . Exploring dengue genome to construct a multi-epitope based subunit vaccine by utilizing immunoinformatics approach to battle against dengue infection. Sci Rep. (2017) 7:9232. doi: 10.1038/s41598-017-09199-w, PMID: 28835708 PMC5569093

[15] Negahdaripour M Nezafat N Eslami M Ghoshoon MB Shoolian E Najafipour S . Structural vaccinology considerations for in silico designing of a multi-epitope vaccine. Infect Genet Evol. (2018) 58:96–109. doi: 10.1016/j.meegid.2017.12.008, PMID: 29253673

[16] Khatoon N Pandey RK Prajapati VK . Exploring Leishmania secretory proteins to design B and T cell multi-epitope subunit vaccine using immunoinformatics approach. Sci Rep. (2017) 7:8285. doi: 10.1038/s41598-017-08842-w, PMID: 28811600 PMC5557753

[17] Shey RA Ghogomu SM Esoh KK Nebangwa ND Shintouo CM Nongley NF . In-silico design of a multi-epitope vaccine candidate against onchocerciasis and related filarial diseases. Sci Rep. (2019) 9:4409. doi: 10.1038/s41598-019-40833-x, PMID: 30867498 PMC6416346

[18] Atapour A Negahdaripour M Ghasemi Y Razmjuee D Savardashtaki A Mousavi SM . In silico designing a candidate vaccine against breast cancer. Int J Pept Res Ther. (2020) 26:369–80. doi: 10.1007/s10989-019-09843-1

[19] Lennerz V Gross S Gallerani E Sessa C Mach N Boehm S . Immunologic response to the survivin-derived multi-epitope vaccine EMD640744 in patients with advanced solid tumors. Cancer Immunol Immunother. (2014) 63:381–94. doi: 10.1007/s00262-013-1516-5, PMID: 24487961 PMC11029529

[20] Song X Li Y Wu H Qiu H Sun Y . T-cell epitope-based vaccines: A promising strategy for prevention of infectious diseases. Vaccines (Basel). (2024) 12:1181. doi: 10.3390/vaccines12101181, PMID: 39460347 PMC11511246

[21] Stop TB Partnership . Available online at: https://newtbvaccines.org/tb-vaccine-pipeline/clinical-phase/ (Accessed September 22, 2025).

[22] Bisht D Meena LS . Adhesion molecules facilitate host-pathogen interaction & mediate Mycobacterium tuberculosis pathogenesis. Indian J Med Res. (2019) 150:23–32. doi: 10.4103/ijmr.IJMR_2055_16, PMID: 31571626 PMC6798602

[23] Yang H Ma Y Lei X Chai S Zhang S Su G . Stopping Tuberculosis at the Gate: The Role of M. tuberculosis Adhesins in Infection and Intervention. Vaccines (Basel). (2025) 13:676. doi: 10.3390/vaccines13070676, PMID: 40733653 PMC12298959

[24] Abou-Zeid C Ratliff TL Wiker HG Harboe M Bennedsen J Rook GA . Characterization of fibronectin-binding antigens released by Mycobacterium tuberculosis and Mycobacterium bovis BCG. Infect Immun. (1988) 56:3046–51. doi: 10.1128/iai.56.12.3046-3051.1988, PMID: 3141278 PMC259698

[25] Karbalaei Zadeh Babaki M Soleimanpour S Rezaee SA . Antigen 85 complex as a powerful Mycobacterium tuberculosis immunogene: Biology, immune-pathogenicity, applications in diagnosis, and vaccine design. Microb Pathog. (2017) 112:20–9. doi: 10.1016/j.micpath.2017.08.040, PMID: 28942172

[26] Bekker LG Dintwe O Fiore-Gartland A Middelkoop K Hutter J Williams A . A phase 1b randomized study of the safety and immunological responses to vaccination with H4:IC31, H56:IC31, and BCG revaccination in Mycobacterium tuberculosis-uninfected adolescents in Cape Town, South Africa. EClinicalMedicine. (2020) 21:100313. doi: 10.1016/j.eclinm.2020.100313, PMID: 32382714 PMC7201034

[27] Jenum S Tonby K Rueegg CS Rühwald M Kristiansen MP Bang P . A Phase I/II randomized trial of H56:IC31 vaccination and adjunctive cyclooxygenase-2-inhibitor treatment in tuberculosis patients. Nat Commun. (2021) 12:6774. doi: 10.1038/s41467-021-27029-6, PMID: 34811370 PMC8608791

[28] Hickey TB Ziltener HJ Speert DP Stokes RW . Mycobacterium tuberculosis employs Cpn60.2 as an adhesin that binds CD43 on the macrophage surface. Cell Microbiol. (2010) 12:1634–47. doi: 10.1111/j.1462-5822.2010.01496.x, PMID: 20633027

[29] Henderson B Allan E Coates AR . Stress wars: the direct role of host and bacterial molecular chaperones in bacterial infection. Infect Immun. (2006) 74:3693–706. doi: 10.1128/iai.01882-05, PMID: 16790742 PMC1489680

[30] Henderson B Lund PA Coates AR . Multiple moonlighting functions of mycobacterial molecular chaperones. Tuberculosis (Edinb). (2010) 90:119–24. doi: 10.1016/j.tube.2010.01.004, PMID: 20338810

[31] Changhong S Hai Z Limei W Jiaze A Li X Tingfen Z . Therapeutic efficacy of a tuberculosis DNA vaccine encoding heat shock protein 65 of Mycobacterium tuberculosis and the human interleukin 2 fusion gene. Tuberculosis (Edinb). (2009) 89:54–61. doi: 10.1016/j.tube.2008.09.005, PMID: 19056317

[32] Alteri CJ Xicohténcatl-Cortes J Hess S Caballero-Olín G Girón JA Friedman RL . Mycobacterium tuberculosis produces pili during human infection. Proc Natl Acad Sci U.S.A. (2007) 104:5145–50. doi: 10.1073/pnas.0602304104, PMID: 17360408 PMC1817835

[33] Wang X Barnes PF Dobos-Elder KM Townsend JC Chung YT Shams H . ESAT-6 inhibits production of IFN-gamma by Mycobacterium tuberculosis-responsive human T cells. J Immunol. (2009) 182:3668–77. doi: 10.4049/jimmunol.0803579, PMID: 19265145 PMC5488288

[34] Yu X Xie J . Roles and underlying mechanisms of ESAT-6 in the context of Mycobacterium tuberculosis-host interaction from a systems biology perspective. Cell Signal. (2012) 24:1841–6. doi: 10.1016/j.cellsig.2012.05.014, PMID: 22634089

[35] Andersen P Andersen AB Sørensen AL Nagai S . Recall of long-lived immunity to Mycobacterium tuberculosis infection in mice. J Immunol. (1995) 154:3359–72. doi: 10.4049/jimmunol.154.7.3359 7897219

[36] Sørensen AL Nagai S Houen G Andersen P Andersen AB . Purification and characterization of a low-molecular-mass T-cell antigen secreted by Mycobacterium tuberculosis. Infect Immun. (1995) 63:1710–7. doi: 10.1128/iai.63.5.1710-1717.1995, PMID: 7729876 PMC173214

[37] Wu M Li M Yue Y Xu W . DNA vaccine with discontinuous T-cell epitope insertions into HSP65 scaffold as a potential means to improve immunogenicity of multi-epitope Mycobacterium tuberculosis vaccine. Microbiol Immunol. (2016) 60:634–45. doi: 10.1111/1348-0421.12410, PMID: 27531823

[38] Bottai D Brosch R . Mycobacterial PE, PPE and ESX clusters: novel insights into the secretion of these most unusual protein families. Mol Microbiol. (2009) 73:325–8. doi: 10.1111/j.1365-2958.2009.06784.x, PMID: 19602151

[39] Mukhopadhyay S Balaji KN . The PE and PPE proteins of Mycobacterium tuberculosis. Tuberculosis (Edinb). (2011) 91:441–7. doi: 10.1016/j.tube.2011.04.004, PMID: 21527209

[40] Ahmed A Das A Mukhopadhyay S . Immunoregulatory functions and expression patterns of PE/PPE family members: Roles in pathogenicity and impact on anti-tuberculosis vaccine and drug design. IUBMB Life. (2015) 67:414–27. doi: 10.1002/iub.1387, PMID: 26104967

[41] Billeskov R Vingsbo-Lundberg C Andersen P Dietrich J . Induction of CD8 T cells against a novel epitope in TB10.4: correlation with mycobacterial virulence and the presence of a functional region of difference-1. J Immunol. (2007) 179:3973–81. doi: 10.4049/jimmunol.179.6.3973, PMID: 17785835

[42] Majlessi L Rojas MJ Brodin P Leclerc C . CD8+-T-cell responses of Mycobacterium-infected mice to a newly identified major histocompatibility complex class I-restricted epitope shared by proteins of the ESAT-6 family. Infect Immun. (2003) 71:7173–7. doi: 10.1128/iai.71.12.7173-7177.2003, PMID: 14638811 PMC308897

[43] Achkar JM Casadevall A . Antibody-mediated immunity against tuberculosis: implications for vaccine development. Cell Host Microbe. (2013) 13:250–62. doi: 10.1016/j.chom.2013.02.009, PMID: 23498951 PMC3759397

[44] Achkar JM Chan J Casadevall A . Role of B cells and antibodies in acquired immunity against Mycobacterium tuberculosis. Cold Spring Harb Perspect Med. (2014) 5:a018432. doi: 10.1101/cshperspect.a018432, PMID: 25301934 PMC4355258

[45] Kozakiewicz L Phuah J Flynn J Chan J . The role of B cells and humoral immunity in Mycobacterium tuberculosis infection. Adv Exp Med Biol. (2013) 783:225–50. doi: 10.1007/978-1-4614-6111-1_12, PMID: 23468112 PMC4184189

[46] Achkar JM Chan J Casadevall A . B cells and antibodies in the defense against Mycobacterium tuberculosis infection. Immunol Rev. (2015) 264:167–81. doi: 10.1111/imr.12276, PMID: 25703559 PMC4629253

[47] Vordermeier HM Venkataprasad N Harris DP Ivanyi J . Increase of tuberculous infection in the organs of B cell-deficient mice. Clin Exp Immunol. (1996) 106:312–6. doi: 10.1046/j.1365-2249.1996.d01-845.x, PMID: 8918578 PMC2200584

[48] Glatman-Freedman A Casadevall A . Serum therapy for tuberculosis revisited: reappraisal of the role of antibody-mediated immunity against Mycobacterium tuberculosis. Clin Microbiol Rev. (1998) 11:514–32. doi: 10.1128/cmr.11.3.514, PMID: 9665981 PMC88894

[49] Wang Q Nag D Baldwin SL Coler RN Mcnamara RP . Antibodies as key mediators of protection against Mycobacterium tuberculosis. Front Immunol. (2024) 15:1430955. doi: 10.3389/fimmu.2024.1430955, PMID: 39286260 PMC11402706

[50] Liu Y Chen T Zhu Y Furey A Lowary TL Chan J . Features and protective efficacy of human mAbs targeting Mycobacterium tuberculosis arabinomannan. JCI Insight. (2023) 8:e167960. doi: 10.1172/jci.insight.167960, PMID: 37733444 PMC10619501

[51] Minerva M De Maio F Camassa S Battah B Ivana P Manganelli R . Evaluation of PE_PGRS33 as a potential surface target for humoral responses against Mycobacterium tuberculosis. Pathog Dis. (2017) 75. doi: 10.1093/femspd/ftx100, PMID: 28911035

[52] Brennan MJ Delogu G Chen Y Bardarov S Kriakov J Alavi M . Evidence that mycobacterial PE_PGRS proteins are cell surface constituents that influence interactions with other cells. Infect Immun. (2001) 69:7326–33. doi: 10.1128/iai.69.12.7326-7333.2001, PMID: 11705904 PMC98818

[53] Basu S Pathak SK Banerjee A Pathak S Bhattacharyya A Yang Z . Execution of macrophage apoptosis by PE_PGRS33 of Mycobacterium tuberculosis is mediated by Toll-like receptor 2-dependent release of tumor necrosis factor-alpha. J Biol Chem. (2007) 282:1039–50. doi: 10.1074/jbc.M604379200, PMID: 17095513

[54] Delogu G Pusceddu C Bua A Fadda G Brennan MJ Zanetti S . Rv1818c-encoded PE_PGRS protein of Mycobacterium tuberculosis is surface exposed and influences bacterial cell structure. Mol Microbiol. (2004) 52:725–33. doi: 10.1111/j.1365-2958.2004.04007.x, PMID: 15101979

[55] Kramarska E Squeglia F De Maio F Delogu G Berisio R . PE_PGRS33, an important virulence factor of mycobacterium tuberculosis and potential target of host humoral immune response. Cells. (2021) 10:161. doi: 10.3390/cells10010161, PMID: 33467487 PMC7830552

[56] Cohen I Parada C Acosta-Gío E Espitia C . The PGRS domain from PE_PGRS33 of mycobacterium tuberculosis is target of humoral immune response in mice and humans. Front Immunol. (2014) 5:236. doi: 10.3389/fimmu.2014.00236, PMID: 24904584 PMC4033847

[57] Brandt L Oettinger T Holm A Andersen AB Andersen P . Key epitopes on the ESAT-6 antigen recognized in mice during the recall of protective immunity to Mycobacterium tuberculosis. J Immunol. (1996) 157:3527–33. doi: 10.4049/jimmunol.157.8.3527, PMID: 8871652

[58] Kariyone A Tamura T Kano H Iwakura Y Takeda K Akira S . Immunogenicity of Peptide-25 of Ag85B in Th1 development: role of IFN-gamma. Int Immunol. (2003) 15:1183–94. doi: 10.1093/intimm/dxg115, PMID: 13679388

[59] Sayes F Sun L Di Luca M Simeone R Degaiffier N Fiette L . Strong immunogenicity and cross-reactivity of Mycobacterium tuberculosis ESX-5 type VII secretion: encoded PE-PPE proteins predicts vaccine potential. Cell Host Microbe. (2012) 11:352–63. doi: 10.1016/j.chom.2012.03.003, PMID: 22520463

[60] Kolaskar AS Tongaonkar PC . A semi-empirical method for prediction of antigenic determinants on protein antigens. FEBS Lett. (1990) 276:172–4. doi: 10.1016/0014-5793(90)80535-q, PMID: 1702393

[61] Doytchinova IA Flower DR . VaxiJen: a server for prediction of protective antigens, tumour antigens and subunit vaccines. BMC Bioinf. (2007) 8:4. doi: 10.1186/1471-2105-8-4, PMID: 17207271 PMC1780059

[62] Dimitrov I Bangov I Flower DR Doytchinova I . AllerTOP v.2–a server for in silico prediction of allergens. J Mol Model. (2014) 20:2278. doi: 10.1007/s00894-014-2278-5, PMID: 24878803

[63] Gupta S Kapoor P Chaudhary K Gautam A Kumar R Raghava GP . In silico approach for predicting toxicity of peptides and proteins. PloS One. (2013) 8:e73957. doi: 10.1371/journal.pone.0073957, PMID: 24058508 PMC3772798

[64] Gupta S Kapoor P Chaudhary K Gautam A Kumar R Raghava GP . Peptide toxicity prediction. Methods Mol Biol. (2015) 1268:143–57. doi: 10.1007/978-1-4939-2285-7_7, PMID: 25555724

[65] Nilsson JB Kaabinejadian S Yari H Kester MGD Van Balen P Hildebrand WH . Accurate prediction of HLA class II antigen presentation across all loci using tailored data acquisition and refined machine learning. Sci Adv. (2023) 9:eadj6367. doi: 10.1126/sciadv.adj6367, PMID: 38000035 PMC10672173

[66] Nilsson JB Kaabinejadian S Yari H Peters B Barra C Gragert L . Machine learning reveals limited contribution of trans-only encoded variants to the HLA-DQ immunopeptidome. Commun Biol. (2023) 6:442. doi: 10.1038/s42003-023-04749-7, PMID: 37085710 PMC10121683

[67] Kaabinejadian S Barra C Alvarez B Yari H Hildebrand WH Nielsen M . Accurate MHC motif deconvolution of immunopeptidomics data reveals a significant contribution of DRB3, 4 and 5 to the total DR immunopeptidome. Front Immunol. (2022) 13:835454. doi: 10.3389/fimmu.2022.835454, PMID: 35154160 PMC8826445

[68] Reynisson B Alvarez B Paul S Peters B Nielsen M . NetMHCpan-4.1 and NetMHCIIpan-4.0: improved predictions of MHC antigen presentation by concurrent motif deconvolution and integration of MS MHC eluted ligand data. Nucleic Acids Res. (2020) 48:W449–w454. doi: 10.1093/nar/gkaa379, PMID: 32406916 PMC7319546

[69] Jensen KK Andreatta M Marcatili P Buus S Greenbaum JA Yan Z . Improved methods for predicting peptide binding affinity to MHC class II molecules. Immunology. (2018) 154:394–406. doi: 10.1111/imm.12889, PMID: 29315598 PMC6002223

[70] Andreatta M Karosiene E Rasmussen M Stryhn A Buus S Nielsen M . Accurate pan-specific prediction of peptide-MHC class II binding affinity with improved binding core identification. Immunogenetics. (2015) 67:641–50. doi: 10.1007/s00251-015-0873-y, PMID: 26416257 PMC4637192

[71] Dhanda SK Vir P Raghava GP . Designing of interferon-gamma inducing MHC class-II binders. Biol Direct. (2013) 8:30. doi: 10.1186/1745-6150-8-30, PMID: 24304645 PMC4235049

[72] Larsen MV Lundegaard C Lamberth K Buus S Lund O Nielsen M . Large-scale validation of methods for cytotoxic T-lymphocyte epitope prediction. BMC Bioinf. (2007) 8:424. doi: 10.1186/1471-2105-8-424, PMID: 17973982 PMC2194739

[73] Peters B Bulik S Tampe R Van Endert PM Holzhütter HG . Identifying MHC class I epitopes by predicting the TAP transport efficiency of epitope precursors. J Immunol. (2003) 171:1741–9. doi: 10.4049/jimmunol.171.4.1741, PMID: 12902473

[74] Bui HH Sidney J Dinh K Southwood S Newman MJ Sette A . Predicting population coverage of T-cell epitope-based diagnostics and vaccines. BMC Bioinf. (2006) 7:153. doi: 10.1186/1471-2105-7-153, PMID: 16545123 PMC1513259

[75] Ferris LK Mburu YK Mathers AR Fluharty ER Larregina AT Ferris RL . Human beta-defensin 3 induces maturation of human langerhans cell-like dendritic cells: an antimicrobial peptide that functions as an endogenous adjuvant. J Invest Dermatol. (2013) 133:460–8. doi: 10.1038/jid.2012.319, PMID: 22951718 PMC3521079

[76] Aziz S Almajhdi FN Waqas M Ullah I Salim MA Khan NA . Contriving multi-epitope vaccine ensemble for monkeypox disease using an immunoinformatics approach. Front Immunol. (2022) 13:1004804. doi: 10.3389/fimmu.2022.1004804, PMID: 36311762 PMC9606759

[77] Kumar V Kancharla S Kolli P Jena M . Reverse vaccinology approach towards the in-silico multiepitope vaccine development against SARS-CoV-2. F1000Res. (2021) 10:44. doi: 10.12688/f1000research.36371.1, PMID: 33841800 PMC8009247

[78] Moqbel Hassan Alzubaydi N Oun Ali Z Al-Asadi S Al-Kahachi R . Design and characterization of a multi-epitope vaccine targeting Chlamydia abortus using immunoinformatics approach. J Biomol Struct Dyn. (2023) 42:6660–77. doi: 10.1080/07391102.2023.2240891, PMID: 37774751

[79] Komai-Koma M Jones L Ogg GS Xu D Liew FY . TLR2 is expressed on activated T cells as a costimulatory receptor. Proc Natl Acad Sci U.S.A. (2004) 101:3029–34. doi: 10.1073/pnas.0400171101, PMID: 14981245 PMC365739

[80] Zhang L Wei X Zhang R Mozdziak PE Si D Ahmad B . Design and immunological evaluation of a hybrid peptide as a potent TLR2 agonist by structure-based virtual screening. Front Cell Dev Biol. (2021) 9:620370. doi: 10.3389/fcell.2021.620370, PMID: 33644058 PMC7905067

[81] Oliveira-Nascimento L Massari P Wetzler LM . The role of TLR2 in infection and immunity. Front Immunol. (2012) 3:79. doi: 10.3389/fimmu.2012.00079, PMID: 22566960 PMC3342043

[82] Su L Wang Y Wang J Mifune Y Morin MD Jones BT . Structural basis of TLR2/TLR1 activation by the synthetic agonist diprovocim. J Med Chem. (2019) 62:2938–49. doi: 10.1021/acs.jmedchem.8b01583, PMID: 30829478 PMC6537610

[83] Jiang F Han Y Liu Y Xue Y Cheng P Xiao L . A comprehensive approach to developing a multi-epitope vaccine against Mycobacterium tuberculosis: from in silico design to *in vitro* immunization evaluation. Front Immunol. (2023) 14:1280299. doi: 10.3389/fimmu.2023.1280299, PMID: 38022558 PMC10652892

[84] Gudkov AT . The L7/L12 ribosomal domain of the ribosome: structural and functional studies. FEBS Lett. (1997) 407:253–6. doi: 10.1016/s0014-5793(97)00361-x, PMID: 9175862

[85] Gul H Ali SS Saleem S Khan S Khan J Wadood A . Subtractive proteomics and immunoinformatics approaches to explore Bartonella bacilliformis proteome (virulence factors) to design B and T cell multi-epitope subunit vaccine. Infect Genet Evol. (2020) 85:104551. doi: 10.1016/j.meegid.2020.104551, PMID: 32931955

[86] Ropón-Palacios G Chenet-Zuta ME Otazu K Olivos-Ramirez GE Camps I . Novel multi-epitope protein containing conserved epitopes from different Leishmania species as potential vaccine candidate: Integrated immunoinformatics and molecular dynamics approach. Comput Biol Chem. (2019) 83:107157. doi: 10.1016/j.compbiolchem.2019.107157, PMID: 31751887

[87] Sethi G Varghese RP Lakra AK Nayak SS Krishna R Hwang JH . Immunoinformatics and structural aided approach to develop multi-epitope based subunit vaccine against Mycobacterium tuberculosis. Sci Rep. (2024) 14:15923. doi: 10.1038/s41598-024-66858-5, PMID: 38987613 PMC11237054

[88] Ismail S Abbasi SW Yousaf M Ahmad S Muhammad K Waheed Y . Design of a multi-epitopes vaccine against hantaviruses: an immunoinformatics and molecular modelling approach. Vaccines (Basel). (2022) 10:378. doi: 10.3390/vaccines10030378, PMID: 35335010 PMC8953224

[89] Feng GD Xue XC Gao ML Wang XF Shu Z Mu N . Therapeutic effects of PADRE-BAFF autovaccine on rat adjuvant arthritis. BioMed Res Int. (2014) 2014:854954. doi: 10.1155/2014/854954, PMID: 24791002 PMC3984822

[90] Snook AE Baybutt TR Xiang B Abraham TS Flickinger JC Jr. Hyslop T . Split tolerance permits safe Ad5-GUCY2C-PADRE vaccine-induced T-cell responses in colon cancer patients. J Immunother Cancer. (2019) 7:104. doi: 10.1186/s40425-019-0576-2, PMID: 31010434 PMC6477737

[91] Zamani P Teymouri M Nikpoor AR Navashenaq JG Gholizadeh Z Darban SA . Nanoliposomal vaccine containing long multi-epitope peptide E75-AE36 pulsed PADRE-induced effective immune response in mice TUBO model of breast cancer. Eur J Cancer. (2020) 129:80–96. doi: 10.1016/j.ejca.2020.01.010, PMID: 32145473

[92] Arai R Ueda H Kitayama A Kamiya N Nagamune T . Design of the linkers which effectively separate domains of a bifunctional fusion protein. Protein Eng. (2001) 14:529–32. doi: 10.1093/protein/14.8.529, PMID: 11579220

[93] Gul S Ahmad S Ullah A Ismail S Khurram M Tahir Ul Qamar M . Designing a Recombinant Vaccine against Providencia rettgeri Using Immunoinformatics Approach. Vaccines (Basel). (2022) 10:189. doi: 10.3390/vaccines10020189, PMID: 35214648 PMC8876559

[94] Livingston B Crimi C Newman M Higashimoto Y Appella E Sidney J . A rational strategy to design multiepitope immunogens based on multiple Th lymphocyte epitopes. J Immunol. (2002) 168:5499–506. doi: 10.4049/jimmunol.168.11.5499, PMID: 12023344

[95] Wang QM Sun SH Hu ZL Zhou FJ Yin M Xiao CJ . Epitope DNA vaccines against tuberculosis: spacers and ubiquitin modulates cellular immune responses elicited by epitope DNA vaccine. Scand J Immunol. (2004) 60:219–25. doi: 10.1111/j.0300-9475.2004.01442.x, PMID: 15320877

[96] Chen X Zaro JL Shen WC . Fusion protein linkers: property, design and functionality. Adv Drug Delivery Rev. (2013) 65:1357–69. doi: 10.1016/j.addr.2012.09.039, PMID: 23026637 PMC3726540

[97] Wilkins MR Gasteiger E Bairoch A Sanchez JC Williams KL Appel RD . Protein identification and analysis tools in the ExPASy server. Methods Mol Biol. (1999) 112:531–52. doi: 10.1385/1-59259-584-7:531, PMID: 10027275

[98] Jones DT . Protein secondary structure prediction based on position-specific scoring matrices. J Mol Biol. (1999) 292:195–202. doi: 10.1006/jmbi.1999.3091, PMID: 10493868

[99] Garnier J Osguthorpe DJ Robson B . Analysis of the accuracy and implications of simple methods for predicting the secondary structure of globular proteins. J Mol Biol. (1978) 120:97–120. doi: 10.1016/0022-2836(78)90297-8, PMID: 642007

[100] Garnier J Gibrat JF Robson B . GOR method for predicting protein secondary structure from amino acid sequence. Methods Enzymol. (1996) 266:540–53. doi: 10.1016/s0076-6879(96)66034-0, PMID: 8743705

[101] Ko J Park H Heo L Seok C . GalaxyWEB server for protein structure prediction and refinement. Nucleic Acids Res. (2012) 40:W294–7. doi: 10.1093/nar/gks493, PMID: 22649060 PMC3394311

[102] Heo L Park H Seok C . GalaxyRefine: Protein structure refinement driven by side-chain repacking. Nucleic Acids Res. (2013) 41:W384–8. doi: 10.1093/nar/gkt458, PMID: 23737448 PMC3692086

[103] Bowie JU Lüthy R Eisenberg D . A method to identify protein sequences that fold into a known three-dimensional structure. Science. (1991) 253:164–70. doi: 10.1126/science.1853201, PMID: 1853201

[104] Lüthy R Bowie JU Eisenberg D . Assessment of protein models with three-dimensional profiles. Nature. (1992) 356:83–5. doi: 10.1038/356083a0, PMID: 1538787

[105] Laskowski RA Rullmannn JA Macarthur MW Kaptein R Thornton JM . AQUA and PROCHECK-NMR: programs for checking the quality of protein structures solved by NMR. J Biomol NMR. (1996) 8:477–86. doi: 10.1007/bf00228148, PMID: 9008363

[106] Morris AL Macarthur MW Hutchinson EG Thornton JM . Stereochemical quality of protein structure coordinates. Proteins. (1992) 12:345–64. doi: 10.1002/prot.340120407, PMID: 1579569

[107] Wiederstein M Sippl MJ . ProSA-web: interactive web service for the recognition of errors in three-dimensional structures of proteins. Nucleic Acids Res. (2007) 35:W407–10. doi: 10.1093/nar/gkm290, PMID: 17517781 PMC1933241

[108] Berman HM Westbrook J Feng Z Gilliland G Bhat TN Weissig H . The protein data bank. Nucleic Acids Res. (2000) 28:235–42. doi: 10.1093/nar/28.1.235, PMID: 10592235 PMC102472

[109] Desta IT Porter KA Xia B Kozakov D Vajda S . Performance and its limits in rigid body protein-protein docking. Structure. (2020) 28:1071–1081.e3. doi: 10.1016/j.str.2020.06.006, PMID: 32649857 PMC7484347

[110] Vajda S Yueh C Beglov D Bohnuud T Mottarella SE Xia B . New additions to the ClusPro server motivated by CAPRI. Proteins. (2017) 85:435–44. doi: 10.1002/prot.25219, PMID: 27936493 PMC5313348

[111] Kozakov D Hall DR Xia B Porter KA Padhorny D Yueh C . The ClusPro web server for protein-protein docking. Nat Protoc. (2017) 12:255–78. doi: 10.1038/nprot.2016.169, PMID: 28079879 PMC5540229

[112] Kozakov D Beglov D Bohnuud T Mottarella SE Xia B Hall DR . How good is automated protein docking? Proteins. (2013) 81:2159–66. doi: 10.1002/prot.24403, PMID: 23996272 PMC3934018

[113] Weng G Wang E Wang Z Liu H Zhu F Li D . HawkDock: a web server to predict and analyze the protein-protein complex based on computational docking and MM/GBSA. Nucleic Acids Res. (2019) 47:W322–w330. doi: 10.1093/nar/gkz397, PMID: 31106357 PMC6602443

[114] Zacharias M . Protein-protein docking with a reduced protein model accounting for side-chain flexibility. Protein Sci. (2003) 12:1271–82. doi: 10.1110/ps.0239303, PMID: 12761398 PMC2323887

[115] Feng T Chen F Kang Y Sun H Liu H Li D . HawkRank: a new scoring function for protein-protein docking based on weighted energy terms. J Cheminform. (2017) 9:66. doi: 10.1186/s13321-017-0254-7, PMID: 29282565 PMC5745212

[116] Singh A Copeland MM Kundrotas PJ Vakser IA . GRAMM web server for protein docking. Methods Mol Biol. (2024) 2714:101–12. doi: 10.1007/978-1-0716-3441-7_5, PMID: 37676594

[117] Xue LC Rodrigues JP Kastritis PL Bonvin AM Vangone A . PRODIGY: a web server for predicting the binding affinity of protein-protein complexes. Bioinformatics. (2016) 32:3676–8. doi: 10.1093/bioinformatics/btw514, PMID: 27503228

[118] Vangone A Bonvin A . PRODIGY: A contact-based predictor of binding affinity in protein-protein complexes. Bio Protoc. (2017) 7:e2124. doi: 10.21769/BioProtoc.2124, PMID: 34458447 PMC8376549

[119] Abraham MJ Murtola T Schulz R Páll S Smith JC Hess B . GROMACS: High performance molecular simulations through multi-level parallelism from laptops to supercomputers. SoftwareX. (2015) 1–2:19–25. doi: 10.1016/j.softx.2015.06.001

[120] López-Blanco JR Aliaga JI Quintana-Ortí ES Chacón P . iMODS: internal coordinates normal mode analysis server. Nucleic Acids Res. (2014) 42:W271–6. doi: 10.1093/nar/gku339, PMID: 24771341 PMC4086069

[121] Castiglione F Poccia F D'offizi G Bernaschi M . Mutation, fitness, viral diversity, and predictive markers of disease progression in a computational model of HIV type 1 infection. AIDS Res Hum Retroviruses. (2004) 20:1314–23. doi: 10.1089/aid.2004.20.1314, PMID: 15650424

[122] Bernaschi M Castiglione F . Design and implementation of an immune system simulator. Comput Biol Med. (2001) 31:303–31. doi: 10.1016/s0010-4825(01)00011-7, PMID: 11535199

[123] Abdi S Ali A Sayed SF Abutahir Ali A Alam P . Multi-epitope-based vaccine candidate for monkeypox: an in silico approach. Vaccines (Basel). (2022) 10:1564. doi: 10.3390/vaccines10091564, PMID: 36146643 PMC9504424

[124] Morgan RN Ismail NSM Alshahrani MY Aboshanab KM . Multi-epitope peptide vaccines targeting dengue virus serotype 2 created via immunoinformatic analysis. Sci Rep. (2024) 14:17645. doi: 10.1038/s41598-024-67553-1, PMID: 39085250 PMC11291903

[125] Arumugam S Varamballi P . In-silico design of envelope based multi-epitope vaccine candidate against Kyasanur forest disease virus. Sci Rep. (2021) 11:17118. doi: 10.1038/s41598-021-94488-8, PMID: 34429443 PMC8384868

[126] Waqas M Aziz S Liò P Khan Y Ali A Iqbal A . Immunoinformatics design of multivalent epitope vaccine against monkeypox virus and its variants using membrane-bound, enveloped, and extracellular proteins as targets. Front Immunol. (2023) 14:1091941. doi: 10.3389/fimmu.2023.1091941, PMID: 36776835 PMC9908764

[127] Chen Y Song X Chen W Zhao X Yang L Liu D . Epitope screening and self-assembled nanovaccine molecule design of PDCoV-S protein based on immunoinformatics. Front Microbiol. (2024) 15:1402963. doi: 10.3389/fmicb.2024.1402963, PMID: 38903798 PMC11186991

[128] Naveed M Yaseen AR Khalid H Ali U Rabaan AA Garout M . Execution and design of an anti HPIV-1 vaccine with multiple epitopes triggering innate and adaptive immune responses: an immunoinformatic approach. Vaccines (Basel). (2022) 10:869. doi: 10.3390/vaccines10060869, PMID: 35746477 PMC9228812

[129] Carbone A Zinovyev A Képès F . Codon adaptation index as a measure of dominating codon bias. Bioinformatics. (2003) 19:2005–15. doi: 10.1093/bioinformatics/btg272, PMID: 14594704

[130] Grote A Hiller K Scheer M Münch R Nörtemann B Hempel DC . JCat: a novel tool to adapt codon usage of a target gene to its potential expression host. Nucleic Acids Res. (2005) 33:W526–31. doi: 10.1093/nar/gki376, PMID: 15980527 PMC1160137

[131] Liu H Irvine DJ . Guiding principles in the design of molecular bioconjugates for vaccine applications. Bioconjug Chem. (2015) 26:791–801. doi: 10.1021/acs.bioconjchem.5b00103, PMID: 25822926 PMC4694040

[132] Ikai A . Thermostability and aliphatic index of globular proteins. J Biochem. (1980) 88:1895–8., PMID: 7462208

[133] Scherrer S Landolt P Friedel U Stephan R . Distribution and expression of esat-6 and cfp-10 in non-tuberculous mycobacteria isolated from lymph nodes of slaughtered cattle in Switzerland. J Vet Diagn Invest. (2019) 31:217–21. doi: 10.1177/1040638718824074, PMID: 30636533 PMC6838824

[134] Zhang W Shu Q Zhao Z Fan J Lyon CJ Zelazny AM . Antigen 85B peptidomic analysis allows species-specific mycobacterial identification. Clin Proteomics. (2018) 15:1. doi: 10.1186/s12014-017-9177-6, PMID: 29321721 PMC5757288

[135] Zhang Z Dong L Li X Deng T Wang Q . The PE/PPE family proteins of Mycobacterium tuberculosis: evolution, function, and prospects for tuberculosis control. Front Immunol. (2025) 16:1606311. doi: 10.3389/fimmu.2025.1606311, PMID: 40599786 PMC12209268

[136] Gcebe N Michel A Gey Van Pittius NC Rutten V . Comparative genomics and proteomic analysis of four non-tuberculous mycobacterium species and mycobacterium tuberculosis complex: occurrence of shared immunogenic proteins. Front Microbiol. (2016) 7:795. doi: 10.3389/fmicb.2016.00795, PMID: 27375559 PMC4894912

