# Supplementary material for: A multi-epitope vaccine incorporating adhesin-derived antigens protects against Mycobacterium tuberculosis infection and dissemination
Source: Front Immunol. 2025 Nov 19;16:1707471. doi: 10.3389/fimmu.2025.1707471 (PMC12672431; doi:10.3389/fimmu.2025.1707471)
Supplement: Supplementary file 1 [file SupplementaryFile1.pdf]

Supplemental information

A Multi-Epitope Vaccine Incorporating Adhesin-Derived Antigens Protects Against *Mycobacterium tuberculosis* Infection and Dissemination

Haoyan Yang[1,2], Xinkui Lei[1,2], Siyu Chai[1,2], Sigen Zhang[1,2], Guimin Su[1,2*], Lin Du[1,2*]
[1]Research and Development Centre, Beijing Zhifei Lvzhu Biopharmaceutical Co., Ltd., Beijing, China
[2]Beijing Bacterial Vaccine Engineering Research Centre, Beijing, China.
[*]Corresponding author

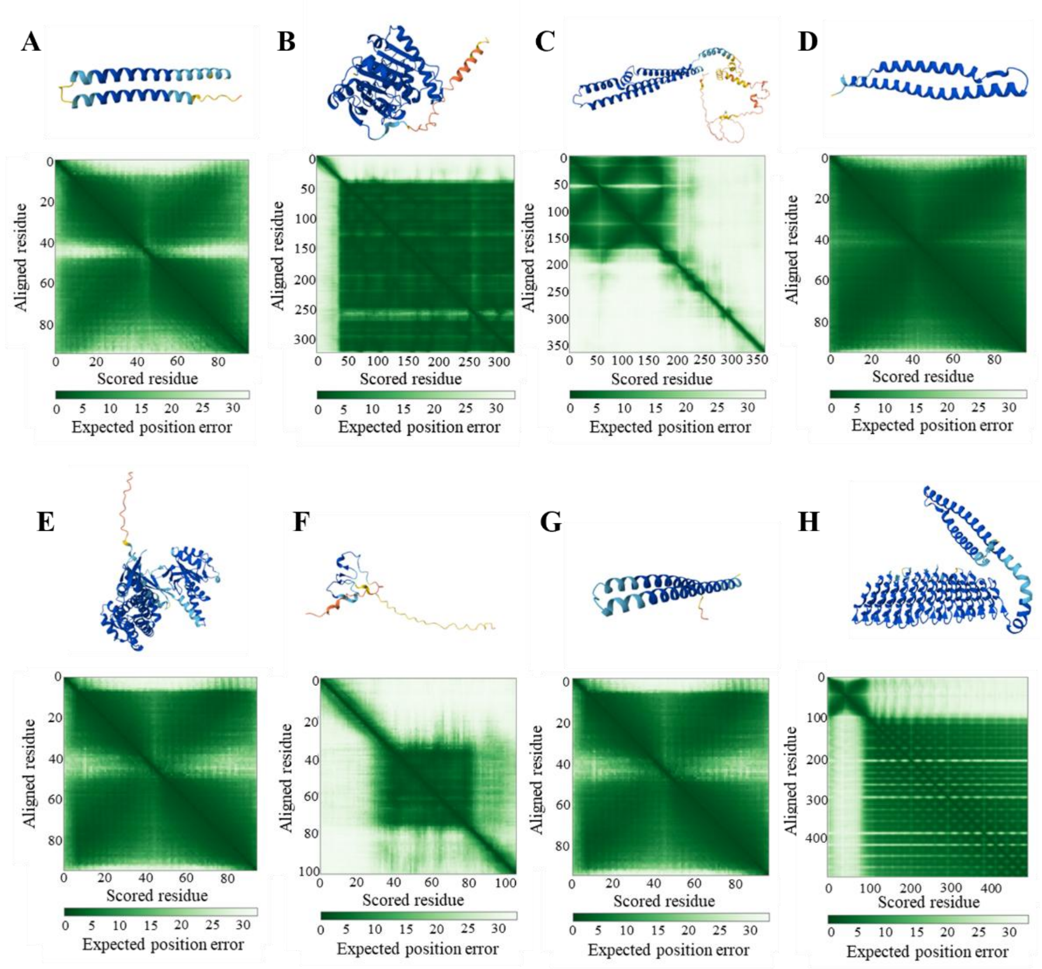

Supplementary Figure 1. Structural analysis of the selected eight proteins by the AlphaFold program. (**A**) ESAT-6, (**B**) Ag85B, (**C**) PPE25, (**D**) PE19 (**E**) HSP65, (**F**) MTP, (**G**) TB10.4, and (**H**) PE_PGRS33.

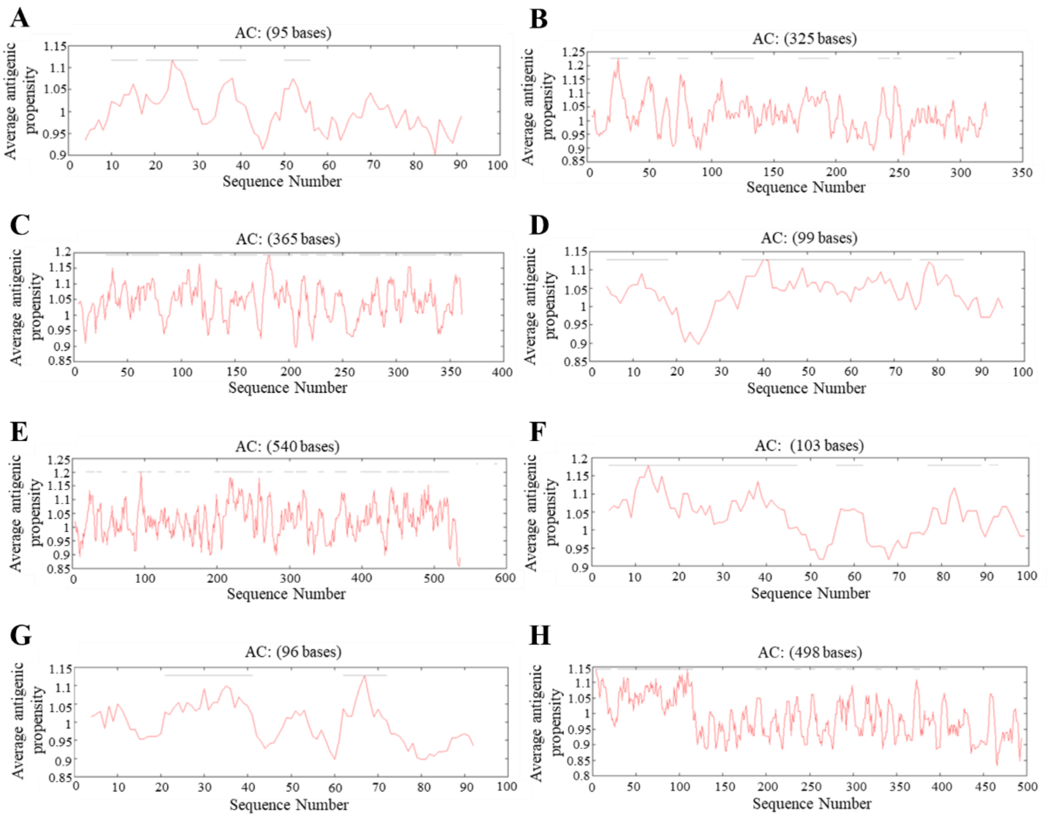

Supplementary Figure 2. The predicted antigenic propensity of the targeted proteins using the Antigenic Peptide Prediction tool is shown as follows: (**A**) ESAT-6, (**B**) Ag85B, (**C**) PPE25, (**D**) PE19 (**E**) HSP65, (**F**) MTP, (**G**) TB10.4, and (**H**) PE_PGRS33.

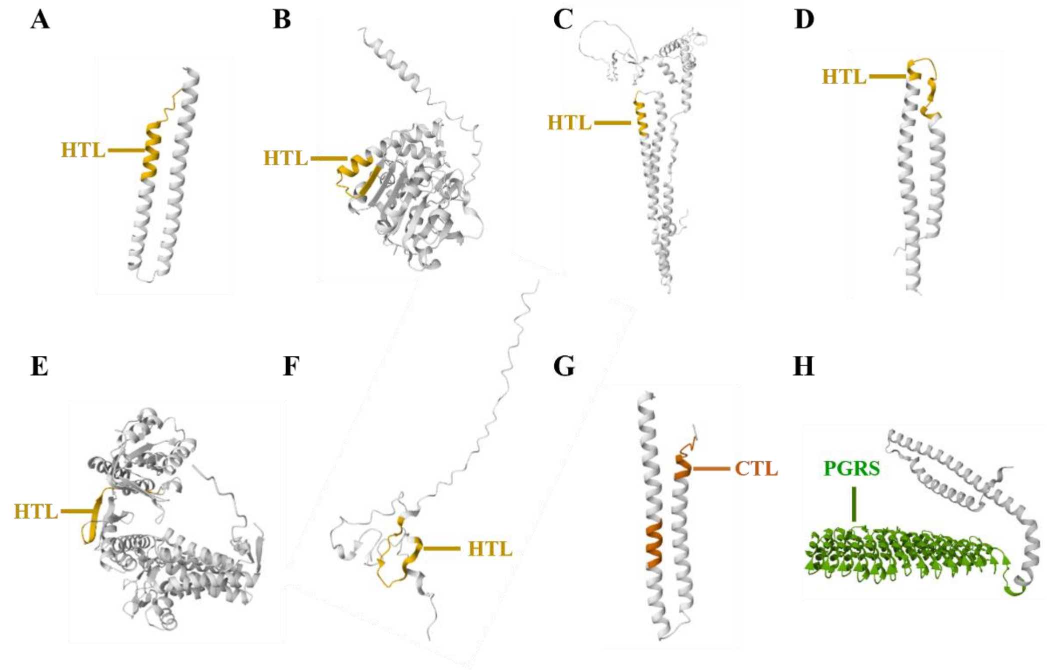

Supplementary Figure 3. The three-dimensional structure predicted by the AlphaFold program for the selected antigenic proteins (**A**) ESAT-6, (**B**) Ag85B, (**C**) PPE25, (**D**) PE19 (**E**) HSP65, (**F**) MTP, (**G**) TB10.4, and (**H**) PE_PGRS33. B-cell epitopes marked in green, whereas HTL and CTL epitopes were marked in light orange and deep orange respectively.

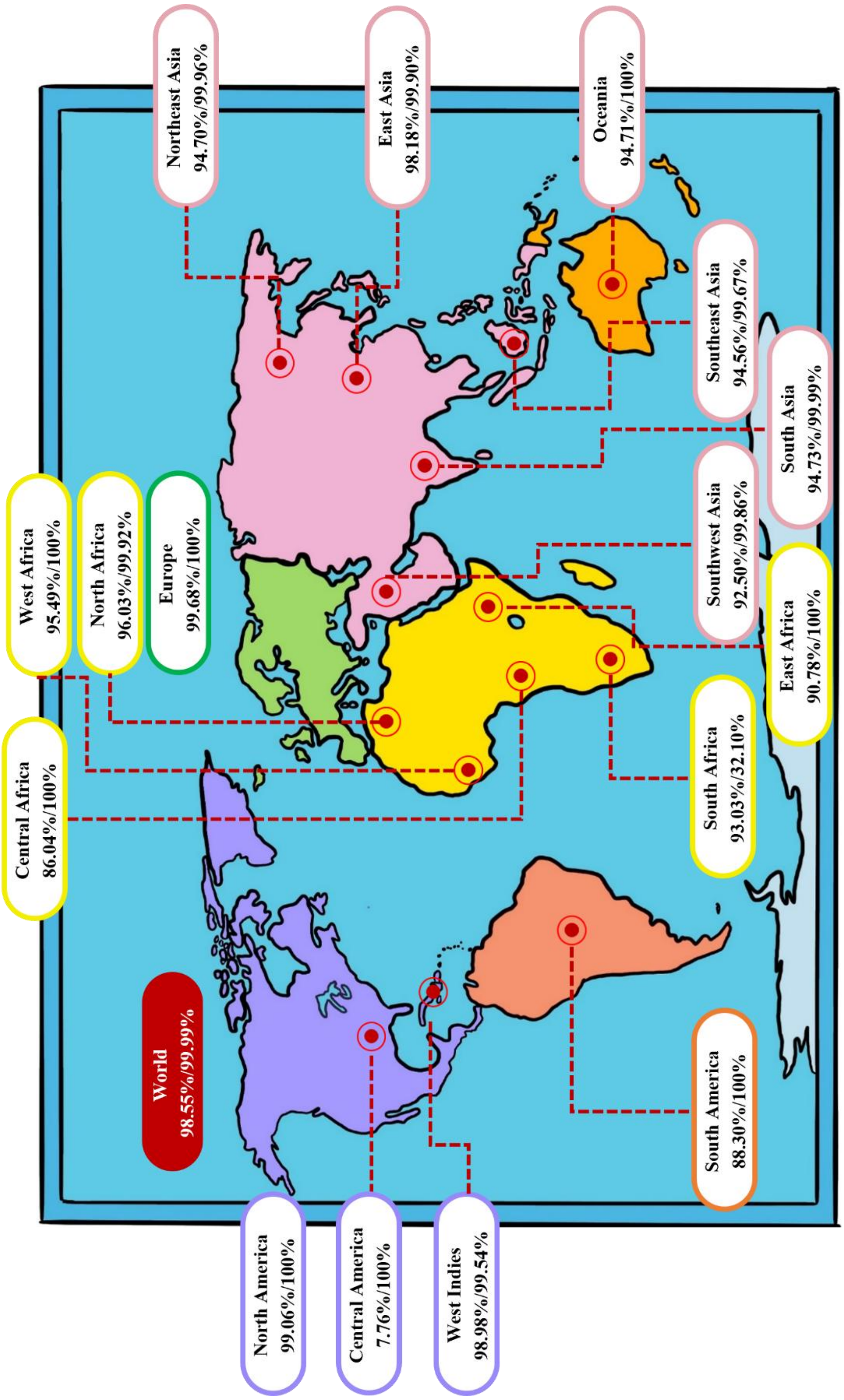

Supplementary Figure 4. Diagrammatic representation of global population coverage of selected epitopes. This map illustrates the global population coverage percentages for MHC class I and MHC class II epitopes across different regions. The coverage values are provided as a range, with MHC class I epitopes on the left and MHC class II epitopes on the right within each regional label.

A

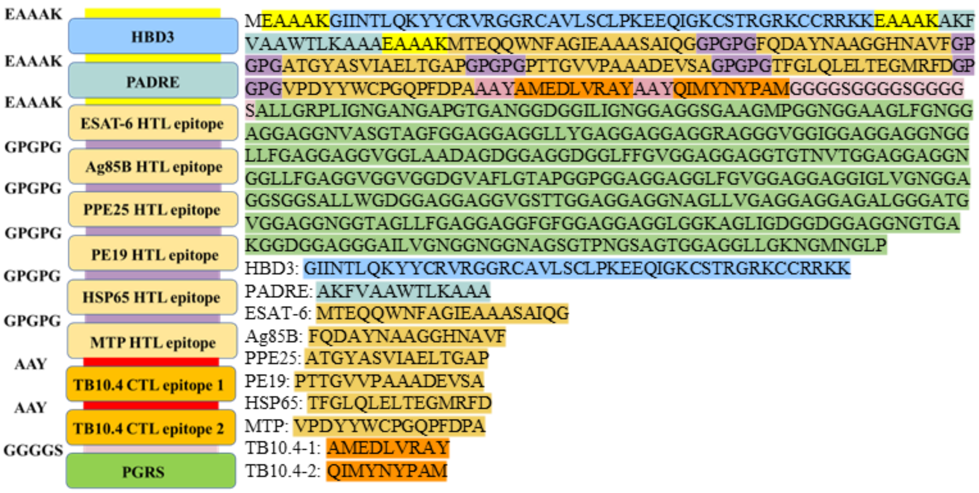

MEAAAKGIINTLQKYYCRVRGGRCAVLSCLPKEEQIGKCSTRGRKCCRRKKEAAAKAKF
VAAWTLKAAAEAAAKMTEQQWNFAGIEAAASAIQGGPGPGFQDAYNAAGGHNAVFGP
GPGATGYASVIAELTGAPGPGPGPTTGVVPAAADEVSAGPGPGTFGLQLELTEGMRFDGP
GPGVPDYYWCPGQPFDPAAAYAMEDLVRAYAAYQIMYNYPAMGGGGSGGGGSGGGG
SALLGRPLIGNGANGAPGTGANGGDGGILIGNGGAGGSGAAGMPGGNGGAAGLFGNGG
AGGAGGNVASGTAGFGGAGGAGGLLYGAGGAGGAGGRAGGGVGGIGGAGGAGGNGG
LLFGAGGAGGVGGLAADAGDGGAGGDGGLFFGVGGAGGAGGTGTNVTGGAGGAGGN
GGLLFGAGGVGGVGGDGVAFLGTAPGGPGGAGGAGGLFGVGGAGGAGGIGLVGNGGA
GGSGGSALLWGDGGAGGAGGVGSTTGGAGGAGGNAGLLVGAGGAGGAGALGGGATG
VGGAGGNGGTAGLLFGAGGAGGFGFGGAGGAGGLGGKAGLIGDGGDGGAGGNGTGA
KGGDGGAGGGAILVGNGGNGGNAGSGTPNGSAGTGGAGGLLGKNGMNGLP

HBD3: GIINTLQKYYCRVRGGRCAVLSCLPKEEQIGKCSTRGRKCCRRKK
PADRE: AKFVAAWTLKAAA
ESAT-6: MTEQQWNFAGIEAAASAIQG
Ag85B: FQDAYNAAGGHNAVF
PPE25: ATGYASVIAELTGAP
PE19: PTTGVVPAAADEVSA
HSP65: TFGLQLELTEGMRFD
MTP: VPDYYWCPGQPFDPA
TB10.4-1: AMEDLVRAY
TB10.4-2: QIMYNYPAM

B

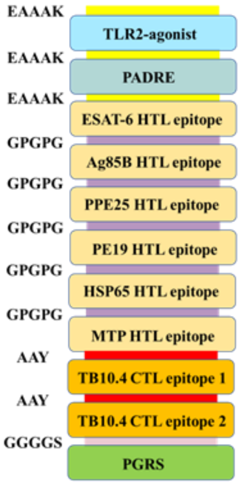

MEAAAKIGKEFKRIVQRIKDFLRNLVPRTERKDVTEAAAKAKFVAAWTLKAAAEAAAKMT
EQQWNFAGIEAAASAIQGGPGPGFQDAYNAAGGHNAVFGPGPGATGYASVIAELTGAPGPG
PGPTTGVVPAAADEVSAGPGPGTFGLQLELTEGMRFDGPGPGVPDYYWCPGQPFDPAAAYA
MEDLVRAYAAYQIMYNYPAMGGGGSGGGGSGGGGSALLGRPLIGNGANGAPGTGANGGD
GGILIGNGGAGGSGAAGMPGGNGGAAGLFGNGGAGGAGGNVASGTAGFGGAGGAGGLLY
GAGGAGGAGGRAGGGVGGIGGAGGAGGNGGLLFGAGGAGGVGGLAADAGDGGAGGDG
GLFFGVGGAGGAGGTGTNVTGGAGGAGGNGGLLFGAGGVGGVGGDGVAFLGTAPGGPGG
AGGAGGLFGVGGAGGAGGIGLVGNGGAGGSGGSALLWGDGGAGGAGGVGSTTGGAGGA
GGNAGLLVGAGGAGGAGALGGGATGVGGAGGNGGTAGLLFGAGGAGGFGFGGAGGAGG
LGGKAGLIGDGGDGGAGGNGTGAKGGDGGAGGGAILVGNGGNGGNAGSGTPNGSAGTGG
AGGLLGKNGMNGLP

TLR2-agonist: IGKEFKRIVQRIKDFLRNLVPRTERKDVT
PADRE: AKFVAAWTLKAAA
ESAT-6: MTEQQWNFAGIEAAASAIQG
Ag85B: FQDAYNAAGGHNAVF
PPE25: ATGYASVIAELTGAP
PE19: PTTGVVPAAADEVSA
HSP65: TFGLQLELTEGMRFD
MTP: VPDYYWCPGQPFDPA
TB10.4-1: AMEDLVRAY
TB10.4-2: QIMYNYPAM

C

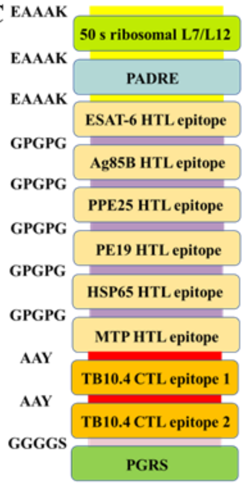

MEAAAKMAKLSTDELLDAFKEMTLLELSDFVKKFEETFEVTAAAPVAVAAAGAAPAGAA
VEAAAEEQSEFDVILEAAGDKKIGVIKVVREIVSGLGLKEAKDLVDGAPKPLLEKVAKEAAD
EAKAKLEAAGATVTVKEAAAKAKFVAAWTLKAAAEAAAKMTEQQWNFAGIEAAASAIQ
GGPGPGFQDAYNAAGGHNAVFGPGPGATGYASVIAELTGAPGPGPGPTTGVVPAAADEVS
AGPGPGTFGLQLELTEGMRFDGPGPGVPDYYWCPGQPFDPAAAYAMEDLVRAYAAYQIM
YNYPAMGGGGSGGGGSGGGGSALLGRPLIGNGANGAPGTGANGGDGGILIGNGGAGGSG
AAGMPGGNGGAAGLFGNGGAGGAGGNVASGTAGFGGAGGAGGLLYGAGGAGGAGGRA
GGGVGGIGGAGGAGGNGGLLFGAGGAGGVGGLAADAGDGGAGGDGGLFFGVGGAGGA
GGTGTNVTGGAGGAGGNGGLLFGAGGVGGVGGDGVAFLGTAPGGPGGAGGAGGLFGVG
GAGGAGGIGLVGNGGAGGSGGSALLWGDGGAGGAGGVGSTTGGAGGAGGNAGLLVGA
GGAGGAGALGGGATGVGGAGGNGGTAGLLFGAGGAGGFGFGGAGGAGGLGGKAGLIG
DGGDGGAGGNGTGAKGGDGGAGGGAILVGNGGNGGNAGSGTPNGSAGTGGAGGLLGK
NGMNGLP

50 s ribosomal L7/L12: MAKLSTDELLDAFKEMTLLELSDFVKKFEETFEVTAAAPVA
VAAAGAAPAGAAVEAAAEEQSEFDVILEAAGDKKIGVIKVVREIVSGLGLKEAKDLVDG
APKPLLEKVAKEAADEAKAKLEAAGATVTVK
PADRE: AKFVAAWTLKAAA
ESAT-6: MTEQQWNFAGIEAAASAIQG
Ag85B: FQDAYNAAGGHNAVF
PPE25: ATGYASVIAELTGAP
PE19: PTTGVVPAAADEVSA
HSP65: TFGLQLELTEGMRFD
MTP: VPDYYWCPGQPFDPA
TB10.4-1: AMEDLVRAY
TB10.4-2: QIMYNYPAM

Supplementary Figure 5. Schematic representation and the primary sequence of the vaccine construct. (**A**) HBD3-vaccine, (**B**) TLR2-vaccine, (**C**) 50 s-vaccine.

A Structure Information

Model	GDT-HA	RMSD	MolProbity	Clash score	Poor rotamers	Rama favored
Initial	1.0000	0.000	2.328	8.1	3.3	91.6
MODEL 1	0.9634	0.400	1.288	4.2	0.4	97.6
MODEL 2	0.9618	0.403	1.303	4.0	0.4	97.4
MODEL 3	0.9545	0.427	1.446	5.7	0.7	97.3
MODEL 4	0.9565	0.429	1.292	3.9	0.4	97.4
MODEL 5	0.9601	0.420	1.382	4.4	0.4	97.1

B Structure Information

Model	GDT-HA	RMSD	MolProbity	Clash score	Poor rotamers	Rama favored
Initial	1.0000	0.000	2.182	7.3	2.6	92.7
MODEL 1	0.9500	0.433	1.245	3.9	0.8	97.7
MODEL 2	0.9459	0.462	1.437	4.3	0.0	96.5
MODEL 3	0.9525	0.431	1.402	4.8	0.0	97.2
MODEL 4	0.9550	0.417	1.262	3.7	0.4	97.5
MODEL 5	0.9554	0.428	1.408	4.2	0.8	96.7

C Structure Information

Model	GDT-HA	RMSD	MolProbity	Clash score	Poor rotamers	Rama favored
Initial	1.0000	0.000	2.192	6.6	3.3	93.3
MODEL 1	0.9653	0.369	1.352	4.4	0.3	97.3
MODEL 2	0.9600	0.392	1.379	4.8	0.3	97.3
MODEL 3	0.9557	0.386	1.282	3.8	0.0	97.4
MODEL 4	0.9614	0.381	1.306	4.4	0.3	97.6
MODEL 5	0.9632	0.383	1.361	4.5	0.3	97.3

Supplementary Figure 6. Detailed evaluation score table for refined models. (**A**) HBD3-vaccine, (**B**) TLR2-vaccine, (**C**) 50 s-vaccine

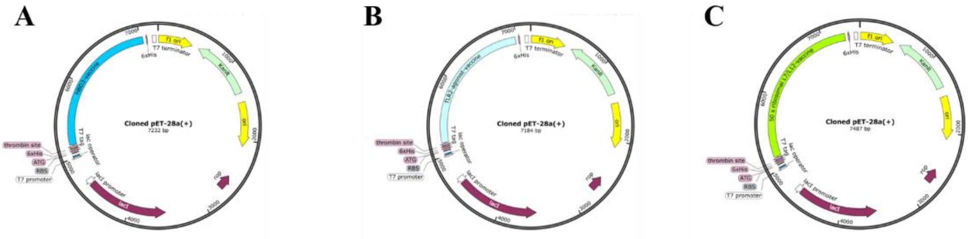

Supplementary Figure 7. Expression vector pET28a (+). In silico restriction cloning of the multi-epitope vaccine sequence into the pET28a (+) expression vector using SnapGene software. (**A**) HBD3-vaccine, (**B**) TLR2-vaccine, (**C**) 50 s-vaccine.

Supplementary Table 1. Selected *M. tuberculosis* proteins used to develop subunit vaccine design.

Physiochemical Properties of Vaccine	Uniprot ID	Antigenic propensity		Protein Length (A.A)	Allergenicity prediction vs. mammal	Toxin
		Immunomedicine Group	VaxiJen 2.0 server			
ESAT-6	P9WNK7	0.9942	0.5577	95	Non-allergen	Non-Toxin
Ag85B	P9WQP1	1.0166	0.5842	325		
PPE25	P9WI13	1.0396	0.4578	365		
PE19	Q79FK4	1.0328	0.6138	99		
HSP65	P9WPE7	1.0251	0.5421	540		
MTP	P9WI87	1.0332	0.7609	103		
TB10.4	P9WNK3	0.9915	0.426	96		
PE_PGRS33	P9WIF5	0.9864	1.6972	498		

Supplementary Table 2. HTL epitopes prediction for the input *M. tuberculosis* protein sequences.

Physiochemical Properties of Vaccine	Protein	Allele	Start	End	Peptide sequence	Percentile score	Method	Result	IFN-γ score
1	ESAT-6	Not available	1	20	MTEQQWNFAGIEAAASAIQG	Not available			
2	Ag85B		280	294	FQDAYNAAGGHNAVF				
3	PPE25	HLA-DRB1*07:01…	42	56	ATGYASVIAELTGAP	0.7414	SVM based	Positive	0.19903727
4	PE19	HLA-DRB4*01:01…	35	49	PTTGVVPAAADEVSA	0.4485	SVM based	Positive	0.22241995
5	HSP65	HLA-DRB1*07:01…	180	194	TFGLQLELTEGMRFD	0.367	SVM based	Positive	0.0921025
6	MTP	HLA-DRB1*07:01…	36	50	VPDYYWCPGQPFDPA	0.3673	SVM based	Positive	0.42145502

Supplementary Table 3. Predicted CTL epitopes from TB10.4 protein sequence of *M. tuberculosis*

Physiochemical Properties of Vaccine	Peptide sequence	Start	End	MHC binding affinity	Rescale binding affinity	C-terminal cleavage affinity	Transport efficiency	Combined Score
1	AMEDLVRAY	61	69	0.3203	1.3601	0.8827	2.997	1.6423
2	QIMYNYPAM	3	11	0.0582	0.247	0.1841	0.565	0.3028

Supplementary Table 4. Table of global population coverage of selected epitopes.

Population/area	Class I			Class II		
	Coverage[a]	Average hit[b]	pc90[c]	Coverage[a]	Average hit[b]	pc90[c]
World	98.55%	18.39	12.11	99.99%	43.27	32.52
Central Africa	86.04%	11.99	5.73	100.00%	44.09	33.66
Central America	7.76%	0.64	0.87	100.00%	37.52	30.21
East Africa	90.78%	13.41	8.18	100.00%	47.7	37.5

East Asia	98.18%	17.83	11.48	99.90%	35.24	24.87
Europe	99.68%	21.46	16.46	100.00%	45.78	35.21
North Africa	96.03%	15.95	9.91	99.92%	34.78	25.13
North America	99.06%	19.58	13.59	100.00%	50.72	41.3
Northeast Aisa	94.70%	14.54	9.19	99.96%	38.23	27.33
Oceania	94.71%	13.3	8.98	100.00%	42.61	32.91
South Africa	93.03%	14.28	8.77	32.10%	2.66	1.18
South America	88.30%	11.68	6.84	100.00%	47.76	37.84
South Asia	94.73%	14.55	9.24	99.99%	41.7	31.57
Southeast Asia	94.56%	14.41	9.14	99.67%	29.42	19.61
Southwest Asia	92.50%	13.81	8.6	99.86%	31.06	21.82
West Africa	95.49%	16.07	9.77	100.00%	44.77	34.58
West Indies	98.98%	20	13.99	99.54%	26.05	17.86

[a] Projected population coverage.

[b] Average number of epitope hits/HLA combinations recognized by the population.

[c] Minimum number of epitope hits/HLA combinations recognized by 90% of the population.

Supplementary Table 5 Physiochemical properties of the determined vaccine.

Physiochemical Properties of Vaccine	HBD3-vaccine	TLR2-agonist-vaccine	50 s -vaccine
Number of amino acids	621	605	706
Molecular weight	53648.62	52043.65	61927.91
Theoretical pI	5.56	4.74	4.42
Total number of negatively charged residues (Asp + Glu)	27	29	50
Total number of positively charged residues (Arg + Lys)	25	21	28
Total number of atoms	7331	7121	8533
Instability index	23.28	21.37	21.34
Aliphatic index	60.77	62.05	68.29
GRAVY	0.12	0.137	0.183
Solubility	0.969421	0.992917	0.798146
Allergenicity vs. mammal (AllerTOP)	Non-allergen	Non-allergen	Non-allergen
Antigenicity (VaxiJen)	1.6944	1.6729	1.5185

Supplementary Table 6. Results of the study of the second structure of the determined vaccine.

Name of the examined unit	HBD3-vaccine		TLR2-vaccine		50 s-vaccine	
	Number of residues	Percentage (%)	Number of residues	Percentage (%)	Number of residues	Percentage (%)
Alpha helix	82	13.20	93	15.37	176	24.93
3_{10} helix	0	0.00	0	0.00	0	0.00
Pi helix	0	0.00	0	0.00	0	0.00
Beta bridge	0	0.00	0	0.00	0	0.00
Extended strand	169	27.21	158	26.12	169	23.94
Beta turn	0	0.00	0	0.00	0	0.00

Bend region	0	0.00	0	0.00	0	0.00
Random coil	370	59.58	354	58.51	361	51.13
Ambiguous states	0	0.00	0	0.00	0	0.00
Other states	0	0.00	0	0.00	0	0.00

Supplementary Table 7. The PRODIGY scoring results of the determined vaccine.

Physiochemical Properties of Vaccine	ΔG (kcal mol-1)	Kd (M) at ℃	ICs charged-charged	ICs charged-polar	ICs charged-apolar	ICs polar-polar	ICs polar-apolar	ICs apolar-apolar	NIS charged	NIS apolar
HawkDock HBD3 model_1	-11.7	2.8E-09	5	13	21	12	37	14	17.06	56.43
HawkDock HBD3 model_2	-8.2	1.1E-06	12	24	30	3	7	8	16.63	56.92
HawkDock HBD3 model_3	-14	5.4E-11	12	12	30	2	32	18	16.78	56.87
GRAMM HBD3 model_1	-28.2	2.1E-21	11	21	108	3	61	51	16.86	56.21
GRAMM HBD3 model_2	-36.8	1E-27	11	19	98	11	110	88	17.5	55.48
GRAMM HBD3 model_3	-37.9	1.7E-28	21	12	129	1	88	59	16.88	55.65
ClusPro 2.0 HBD3 model_1	-16.4	9.8E-13	27	21	59	0	21	50	16.37	56.37
ClusPro 2.0 HBD3 model_2	-15.7	3E-12	24	10	31	4	35	32	16.78	55.86
ClusPro 2.0 HBD3 model_3	-10	4.6E-08	3	5	31	0	15	48	17.18	55.44
HawkDock TLR2 model_1	-18.2	4.1E-14	2	17	61	10	48	27	17.17	56.43
HawkDock TLR2 model_2	-16.9	4.2E-13	3	11	20	7	57	13	17.01	56.48
HawkDock TLR2 model_3	-10.1	3.8E-08	3	18	24	12	30	35	16.78	57.01
GRAMM TLR2 model_1	-37.4	3.6E-28	10	19	138	12	96	57	17.34	55.19
GRAMM TLR2 model_2	-38.1	1.2E-28	15	23	131	21	108	71	16.95	55.81
GRAMM TLR2 model_3	-37.3	4.7E-28	12	30	112	12	106	47	17.09	55.39
ClusPro 2.0 TLR2 model_1	-13.2	2E-10	3	5	41	3	28	38	17.03	56.32
ClusPro 2.0 TLR2 model_2	-6.1	3.1E-05	2	8	12	6	13	42	16.84	56.93

ClusPro 2.0 TLR2 model_3	-10.5	1.9E-08	1	4	14	2	28	40	17.11	56.18
HawkDock 50 s model_1	-13.6	9.9E-11	5	14	43	5	31	11	18.32	56.74
HawkDock 50 s model_2	-18.1	5E-14	2	19	58	9	49	28	18.32	56.87
HawkDock 50 s model_3	-12.2	1.2E-09	1	9	29	14	40	19	18.58	56.37
GRAMM 50 s model_1	-49.5	4.6E-37	7	15	105	24	178	85	18.56	57.1
GRAMM 50 s model_2	-42.8	4.1E-32	37	40	147	11	106	124	17.79	57.7
GRAMM 50 s model_3	-26.7	2.4E-20	44	42	74	11	65	60	17.52	58.49
ClusPro 2.0 50 s model_1	-12.1	1.4E-09	2	8	35	3	27	74	18.49	56.43
ClusPro 2.0 50 s model_2	-13.4	1.5E-10	5	16	40	7	33	72	18.53	56.55
ClusPro 2.0 50 s model_3	-9.7	7.3E-08	8	11	28	1	16	53	18.14	57.25

Supplementary Table 8. MM-PBSA values of the determined vaccine.

Delta Energy Terms

HBD3-vaccine			
**Energy Component**	**Average**	**Std Dev**	**Err. of Mean**
Van der Waals Energy	-236.53	27.06	0.27
Electrostatic Energy	-1087.90	285.50	2.83
Gas-Phase Energy	-1324.43	307.83	3.05
Solvation Energy	1266.63	291.14	2.88
Total	-57.80	24.19	0.24
**TLR2-vaccine**			
**Energy Component**	**Average**	**Std Dev**	**Err. of Mean**
Van der Waals Energy	-116.40	60.32	0.60
Electrostatic Energy	332.26	107.20	1.06
Gas-Phase Energy	215.87	158.38	1.57
Solvation Energy	-198.08	162.61	1.61
Total	17.78	18.64	0.18
**50 s-vaccine**			
**Energy Component**	**Average**	**Std Dev**	**Err. of Mean**

Van der Waals Energy	268.00	37.45	0.37
Electrostatic Energy	942.83	126.32	1.25
Gas-Phase Energy	1210.83	134.08	1.33
Solvation Energy	-777.42	130.53	1.29
Total	433.40	24.31	0.24

Supplementary Table 9. Summary of online servers and tools used in this study

Physiochemical Properties of Vaccine	Description	Uniform Resource Locator (URL)
Uniprot	Resource of protein sequence and functional information	
Protein Databank (PDB)	Resource of protein structure and functional information	https://www.rcsb.org
Immunomedicine Group	Prediction of antigenicity	http://imed.med.ucm.es/Tools/antigenic.pl
VaxiJen 2.0	Prediction of antigenicity	http://www.ddg-pharmfac.net/vaxijen/VaxiJen/VaxiJen.html
AllerTOP v. 2.0	Prediction of allergenicity	https://www.ddg-pharmfac.net/AllerTOP/
ToxinPred	Prediction of toxic/non-toxic peptides	https://webs.iiitd.edu.in/raghava/toxinpred/index.html
IEDB MHC II	Prediction of Helper T lymphocytes epitope	http://tools.iedb.org/mhcii/
IFNepitope	Prediction of interferon-gamma inducing epitopes	http://crdd.osdd.net/raghava/ifnepitope/predict.php
NetCTL 1.2	Prediction of cytotoxic T lymphocytes epitope	https://services.healthtech.dtu.dk/services/NetCTL-1.2/
AlphaFold Protein Structure Database	Tertiary structure prediction	https://alphafold.ebi.ac.uk/
IEDB Population Coverage	Population coverage	http://tools.iedb.org/population/
ProtParam	Physiochemical properties	https://web.expasy.org/protparam/
SOLPro	Solubility prediction	https://scratch.proteomics.ics.uci.edu/
PRISPRED	Secondary structure prediction	http://bioinf.cs.ucl.ac.uk/psipred/
Prabi	Secondary structure prediction	https://npsa-prabi.ibcp.fr/cgi-bin/npsa_automat.pl?page=npsa_gor4.html
AlphaFold Server	Tertiary structure prediction	https://golgi.sandbox.google.com/about
GalaxyRefine	Tertiary structure refinement	http://galaxy.seoklab.org/cgi-bin/submit.cgi?type=REFINE
SAVES v6.1	Structure validation	https://saves.mbi.ucla.edu/
ProSA-web	Structure validation	https://prosa.services.came.sbg.ac.at/prosa.php
PyMOL	Molecular visualization system	https://pymol.org/
ClusPro 2.0	Molecular docking	https://cluspro.bu.edu/login.php
HawkDock	Molecular docking	http://cadd.zju.edu.cn/hawkdock/
GRAMM	Molecular docking	https://gramm.compbio.ku.edu/request
PRODIGY	Prediction of binding affinity in biological complexes	https://rascar.science.uu.nl/prodigy/
LigPlot+	Analysis of molecular interactions in complexes	https://www.ebi.ac.uk/thornton-srv/software/LigPlus/download2.html
GROMACS	Molecular dynamic simulation	https://www.gromacs.org/
iMODS	Molecular dynamics simulation	http://imods.Chaconlab.org/
JCat	Codon optimization	https://www.jcat.de/
SnapGene	*In-silico* simulation	https://www.snapgene.com/
C-ImmSim	Immune simulation	https://kraken.iac.rm.cnr.it/C-IMMSIM/index.php
GraphPad	Statistical analysis	www.graphpad.com
